# Dedifferentiation of smooth muscle cells in intracranial aneurysms and its potential contribution to the pathogenesis

**DOI:** 10.1038/s41598-020-65361-x

**Published:** 2020-05-20

**Authors:** Mieko Oka, Satoshi Shimo, Nobuhiko Ohno, Hirohiko Imai, Yu Abekura, Hirokazu Koseki, Haruka Miyata, Kampei Shimizu, Mika Kushamae, Isao Ono, Kazuhiko Nozaki, Akitsugu Kawashima, Takakazu Kawamata, Tomohiro Aoki

**Affiliations:** 1 Department of Molecular Pharmacology, Research Institute, National Cerebral and Cardiovascular Center, 6-1 Kishibeshinmachi, Suita City, Osaka, 564-8565 Japan; 2 Core Research for Evolutional Science and Technology from Japan Agency for Medical Research and Development, National Cerebral and Cardiovascular Center, 6-1 Kishibeshinmachi, Suita City, Osaka, 564-8565 Japan; 3 Department of Neurosurgery, Tokyo Women’s Medical University, 8-1 Kawata-cho, Shinjyuku-ku, Tokyo, 162-8666 Japan; 4 Department of Occupational Therapy, Health Science University, 7181 Kodachi, Minamitsurugun Fujikawaguchikomachi, Yamanashi, 401-0380 Japan; 5 Division of Histology and Cell Biology, Department of Anatomy, Jichi Medical University, 3311-1 Yakushiji, Shimotsuke City, Tochigi, 329-0498 Japan; 6 Division of Ultrastructural Research, National Institute for Physiological Sciences, 38 Saigonaka, Meidaiji-cho, Okazaki City, Aichi 444-8787 Japan; 7 Department of Systems Science, Graduate School of Informatics, Kyoto University, 36-1 Yoshidahomachi Saikyo-ku, Kyoto City, Kyoto, 606-8317 Japan; 8 Department of Neurosurgery, Kyoto University Graduate School of Medicine, 54 Kawahara-cho Shogoin, Sakyo-ku, Kyoto, 606-8507 Japan; 9 Department of Neurosurgery, The Jikei University School of Medicine, 3-25-8 Nishi-Shimbashi, Minato-ku, Tokyo, 105-8461 Japan; 10 Department of Neurosurgery, Shiga University of Medical Science, Seta Tsukinowa-cho, Otsu City, Shiga, 520-2192 Japan; 11 Department of Neurosurgery, Showa University, 1-5-8 Hatanodai, Shinagawa-ku, Tokyo, 142-8666 Japan; 12 Department of Neurosurgery, Tokyo Women’s Medical University Yachiyo Medical Center, 477-96 Oowadashinden, Yachiyo City, Chiba, 276-8524 Japan

**Keywords:** Cardiovascular biology, Aneurysm

## Abstract

Smooth muscle cells (SMCs) are the major type of cells constituting arterial walls and play a role to maintain stiffness via producing extracellular matrix. Here, the loss and degenerative changes of SMCs become the major histopathological features of an intracranial aneurysm (IA), a major cause of subarachnoid hemorrhage. Considering the important role of SMCs and the loss of this type of cells in IA lesions, we in the present study subjected rats to IA models and examined how SMCs behave during disease progression. We found that, at the neck portion of IAs, SMCs accumulated underneath the internal elastic lamina according to disease progression and formed the intimal hyperplasia. As these SMCs were positive for a dedifferentiation marker, myosin heavy chain 10, and contained abundant mitochondria and rough endoplasmic reticulum, SMCs at the intimal hyperplasia were dedifferentiated and activated. Furthermore, dedifferentiated SMCs expressed some pro-inflammatory factors, suggesting the role in the formation of inflammatory microenvironment to promote the disease. Intriguingly, some SMCs at the intimal hyperplasia were positive for CD68 and contained lipid depositions, indicating similarity with atherosclerosis. We next examined a potential factor mediating dedifferentiation and recruitment of SMCs. Platelet derived growth factor (PDGF)-BB was expressed in endothelial cells at the neck portion of lesions where high wall shear stress (WSS) was loaded. PDGF-BB facilitated migration of SMCs across matrigel-coated pores in a transwell system, promoted dedifferentiation of SMCs and induced expression of pro-inflammatory genes in these cells *in vitro*. Because, in a stenosis model of rats, PDGF-BB expression was expressed in endothelial cells loaded in high WSS regions, and SMCs present nearby were dedifferentiated, hence a correlation existed between high WSS, PDGFB and dedifferentiation *in vivo*. In conclusion, dedifferentiated SMCs presumably by PDGF-BB produced from high WSS-loaded endothelial cells accumulate in the intimal hyperplasia to form inflammatory microenvironment leading to the progression of the disease.

## Introduction

One of the major histopathological characters of intracranial aneurysms (IAs) is excessive degenerative changes in the media including loss, disorganization and non-physiological stretch of medial smooth muscle cells (SMCs)^1–4^. For example, in the histopathological study enrolling IA lesions from total 66 cases (42 cases with ruptured IA and 24 cases with unruptured lesion), almost half of IA lesions has the hypo-cellular wall and most of lesions with such a hypo-cellular wall is ruptured one^5^ Similarly, the histopathological study has revealed the endothelial damage, fewer smooth muscle α-actin (SMA)-positive cells and inflammatory cell invasion as findings predominantly observed in ruptured lesions^6^. Another histopathological study enrolling IA lesions from total 37 cases (17 cases with ruptured IA and 30 cases with unruptured one) has demonstrated that the area containing SMA-positive SMCs in ruptured lesions is significantly smaller than that in unruptured IA lesions^3^. SMC constitutes a major fraction of an arterial wall and functions to maintain stiffness of arterial walls through producing extracellular matrix, mainly collagens. The loss of medial SMCs and resultant decrease of extracellular matrix production thus leads to remarkable reduction in stiffness of arterial walls, presumably making IAs being more fragile and rupture-prone and leading to the devastating outcome due to subarachnoid hemorrhage (SAH). The study analyzing a course of sodium dodecyl sulfate-induced decellularized saccular aneurysms in rats may support above assumption. In this study, decellularized lesions follow more unstable course than non-decellularized ones and only lesions in decellularized group rupture during the observation period^7^. We therefore examined the change of SMCs over time and the contribution to the pathological condition during the progression of IA lesions using a rat model.

## Materials and Methods

### Rodent IA models and histological analysis of induced IA

All of the following experiments including animal care and use complied with the National Institutes of Health Guide for the Care and Use of Laboratory Animals and the Animal Research Reporting *In Vivo* Experiments (ARRIVE) guidelines and were approved by the Institutional Animal Care and Use Committee of National Cerebral and Cardiovascular Center (approval number #17085, #18010 and #19036) and of Kyoto University (#19008).

Sprague-Dawley rats were purchased from Japan SLC (Shizuoka, Japan). Animals were maintained on a light/dark cycle of 12 h/12 h and had free access to chow and water. To induce IA, male or female rats at 7-week-old were subjected to the ligation of the left carotid artery, and systemic hypertension induced by salt overloading and the ligation of left renal artery under general anesthesia by the intraperitoneal injection of pentobarbital sodium (60 mg/kg) and the inhalation of Isoflurane (1.5~2.5%)^8–10^. This procedure to induce IAs is designed to increase the hemodynamic stress, the putative trigger of IA formation^11–13^, loaded on bifurcation sites in right half of intracranial arteries. From just after surgical manipulations above, animals were fed the special chow containing 8% sodium chloride and 0.12% 3-aminopropionitrile (Tokyo chemical industry, Tokyo, Japan), an irreversible and a specific inhibitor of Lysyl Oxidase that catalyzes the cross-linking of collagen and elastin. IA induction at right anterior cerebral artery (ACA) and olfactory artery (OA) bifurcation was assessed at the indicated time in the ‘Results section’ or the ‘Figure Legends’ after surgical manipulations. In immunohistochemical or electron microscopic analyses, animals were deeply anesthetized by the intraperitoneal injection of a lethal dose of pentobarbital sodium (200 mg/kg) and transcardially perfused with 4% paraformaldehyde (PFA) solution.

### Immunohistochemistry

At the indicated period after surgical manipulations, 5-µm-thick frozen sections were prepared. After blocking with 3% donkey serum (Jackson ImmunoResearch, Baltimore, MD), slices were incubated with primary antibodies followed by incubation with secondary antibodies conjugated with a fluorescence dye (Thermo Fisher Scientific, Waltham, MA). Finally, fluorescent images were acquired on a confocal fluorescence microscope system (FV1000, Olympus, Tokyo, Japan). The area of positive signals in immunohistochemistry was quantified by ImageJ software (https://imagej.nih.gov/ij/index.html).

The following primary antibodies were used; mouse monoclonal anti-smooth muscle alpha actin (SMA) antibody (#M0851, Dako, Agilent, Santa Clara, CA), mouse monoclonal anti-non-muscle Myosin IIB/myosin heavy chain 10 (MYH10) antibody (#ab684, Abcam, Cambridge, UK), rabbit polyclonal anti-tumor necrosis factor (TNF)-alpha antibody (#ab6671, Abcam), rabbit polyclonal anti-monocyte chemoattractant protein-1 (MCP-1) antibody (#ab9779, Abcam), mouse polyclonal anti-cyclooxygenase-2 (COX-2) antibody (#aa570–598, Cayman Chemical, Ann Arbor, MI), rabbit polyclonal anti-IL-6 antibody (#ab6672, Abcam), rabbit polyclonal anti-platelet-derived growth factor (PDGF)-AA antibody (#ab198874, Abcam), rabbit polyclonal anti-PDGF-BB antibody (#ab16829, Abcam), rabbit polyclonal anti-Amphiregulin (AREG) antibody (#bs-3847R, Bioss ANTIBODIES, Boston, MA), rabbit polyclonal anti-VE-cadherin (Cadherin 5) antibody (#36–1900, Thermo Fisher Scientific), mouse monoclonal anti-CD68 antibody (#ab31630, Abcam), rabbit polyclonal anti-NFE2L2 antibody (#16396-1-AP, Thermo Fisher Scientific).

The following secondary antibodies were used; Alexa Fluor 488-conjugated donkey anti-mouse IgG H&L antibody (#A21202, Thermo Fisher Scientific), Alexa Fluor 488-conjugated donkey anti-rabbit IgG H&L antibody (#A21206, Thermo Fisher Scientific), Alexa Fluor 594-conjugated donkey anti-mouse IgG H&L antibody (#A21203, Thermo Fisher Scientific), Alexa Fluor 594-conjugated donkey anti-rabbit IgG H&L antibody (#A21207, Thermo Fisher Scientific).

### Serial block-face scanning electron microscopy (SBF-SEM)

Histological analysis with SBF-SEM and processing of acquired data were performed as described previously^14,15^. Arterial samples at the right ACA-OA bifurcation site including induced IA lesions in a rat model were fixed in 2% glutaraldehyde and 2% PFA in 0.1 M phosphate buffer (pH 7.4) at 4 °C overnight, treated with 2% OsO_4_ and 1.5% potassium ferricyanide in phosphate buffered saline for additional 1 h at 4 °C, 1% thiocarbohydrazide for 20 min at room temperature, 2% aqueous OsO_4_ for 30 min at room temperature and the lead aspartate solution for 30 min at 65 °C. The samples were then dehydrated in graded ethanol series, treated with dehydrated acetone, and embedded in Quetol 812 epoxy resin (Nisshin EM Co., Tokyo, Japan) containing Ketjen black powder for 3 nights at 70 °C for polymerization^16^. SBF-SEM observation was performed using a Merlin scanning electron microscope (Carl Zeiss, Gottingen, Germany) equipped with a 3View in-chamber ultra-microtome system (Gatan, Pleasanton, CA). Serial image sequences were 221 µm × 221 µm wide (12 nm/pixel) and over 100 µm deep at 100 nm steps. The sequential images were processed using a FIJI (https://fiji.sc/). Segmentation and three-dimensional reconstruction were performed using an Amira (Thermo Fisher Scientific). The volume, the surface area, the center point, and the distance between points were then calculated an Amira (Thermo Fisher Scientific). In images, mitochondria were identified as round-oval intracellular organelles with cristae inside, and rough endoplasmic reticulum (rER) as continuous membranous cisternae with ribosomes on its surface. Cells with more than 0.6 × 10^6^/mm^2^ mitochondria and more than 8% rER area per whole cell area were considered as activated ones (Figure S1).

### Immunoelectron microscopy

Animals were sacrificed as described above. Dissected IA preparations were fixed overnight in 4% PFA solution containing 0.1% glutaraldehyde. 10-μm-thick frozen sections were then prepared. After treatment with 1% H_2_O_2_ solution for 30 minutes and blocking with normal goat serum (Jackson ImmunoResearch), the slices were incubated with the primary antibody targeting SMA (#M0851, Dako) overnight, followed by the incubation with a biotin-labeled secondary antibody (Biotin-conjugated goat anti-mouse IgG H&L antibody, #ab6788, Abcam). The slices were then treated with a VECTASTAIN ABC kit (VECTOR Laboratories, Burlingame, CA) and DAB (3, 3-diaminobenzidine) substrate (VECTOR Laboratories) for color development, followed by the incubation in 1% osmium tetroxide solution. After the dehydration, the slices were embedded in a Quetol 812 epoxy resin (Nisshin EM Co.), and their ultrathin sections at 60 nm thickness were observed with a transmission electron microscopic system (HT7700 transmission electron microscope, HITACHI, Tokyo, Japan).

### Stenosis model of carotid artery of a rat

The left common carotid artery (CCA) of a 7-week-old male Sprague-Dawley rat was ligated by 10-0 nylon thread with 24 gauge needle put on the side of the artery and the stenosis was established by removing only the needle. From just after above manipulation, animals were fed the chow containing 8% sodium chloride and 0.12% 3-aminopropionitrile (Tokyo chemical industry). On the 30^th^ day, animals were deeply anesthetized by the intraperitoneal injection of a lethal dose of pentobarbital sodium (200 mg/kg) and transcardially perfused with 4% PFA solution. The left CCA and the right one as a control were then stripped and subjected to histological and immunohistochemical analyses as described above.

### MRI acquisition

Rats were placed prone in a cradle under the general anesthesia with an inhalation of isoflurane (1%~2.5%). The rat’s head was fixed using ear bars and a tooth bar. Body temperature was maintained between 35.3 and 37.3 °C by a flow of warm air using a heater system.

MR measurements were conducted on a 7-T preclinical scanner (BioSpec 70/20 USR, Bruker BioSpin MRI GmbH, Ettlingen, Germany) with a quadrature transmit-receive volume coil to detect MRI signals (inner diameter 72 mm, #T9562, Bruker BioSpin). MRI data were then acquired with the dedicated operation software (ParaVision (version 5.1), Bruker BioSpin). To obtain morphological information on carotid arteries, three-dimensional time-of-flight MR angiography (MRA) was performed with the following acquisition parameters; flow compensated gradient echo pulse sequence, TR 30 msec, TE 2.52 msec, flip angle 40°, field of view (FOV) 32 × 32 × 16 mm^3^, acquisition matrix size 160 × 160 × 80, isotropic spatial resolution 200 µm, axial orientation, number of averages = 1, and scan time approximately 6.5 minutes.

### Primary culture of SMCs from human carotid artery

The primary culture of SMCs from human carotid artery was purchased from Cell Applications (#3514-05a, San Diego, CA). Cells were maintained in the special medium from the company. Cells within P5 were served to each experiment.

### Transwell assay

The primary culture of SMCs was cultured on a matrigel-coated chamber with 8-μm pore (Corning, One Riverfront Plaza Corning, NY). 100 ng/ml recombinant human PGDF-BB protein (R&D Systems, Minneapolis, MN) or 100 ng/ml recombinant human AREG protein (R&D Systems) was added in a lower chamber and migrated SMCs across pores within 24 h were counted after staining by a Differential Quik Stain Kit (Polysciences Inc., Warrington, PA).

### Quantitative real time-PCR analysis

Primary culture of SMCs was treated with 100 ng/ml recombinant PDGF-BB (R&D Systems) or 100 ng/ml recombinant AREG (R&D Systems) for 1 or 24 h. Some cells were also treated with each concentration of recombinant PDGF-BB (1, 5, 25, 100, 200 ng/ml, R&D Systems) for 1 h. RNA purification from treated cells and reverse transcription were done using a RNeasy Plus Mini Kit (QIAGEN, Hilden, Germany) and a High-capacity cDNA Reverse Transcription Kit (Life Technologies Corporation, Carlsbad, CA). Quantitative real time-PCR was performed for the quantification of gene expression on a Real Time System CFX96 (Bio-rad, Hercules, CA) and a LightCycler 480 (Roche, Basel, Switzerland) with a SYBR Premix Ex Taq II (Takara Bio Inc., Shiga, Japan). For quantification, the second derivative maximum method was used for crossing point determination. Gene expression of β-actin (*ACTB*) was used as an internal control.

Primers used in the present experiment are listed in following; forward 5′-TCAGCAATGAGTGACAGTTGG-3′ and reverse 5′-ATAGGCTGTTCCCATGTAGCC -3′ for *TNF*, forward 5′-AGCTTCTTTGGGACACTTGC-3′ and reverse 5′-ATAGCAGCCACCTTCATTCC-3′ for *CCL2*, forward 5′-ACACCCTCTATCACTGGCATCC-3′ and reverse 5′-AACATTCCTACCACCAGCAACC-3′ for *PTGS2* (a gene encoding COX-2), forward 5′-CATTTGTGGTTGGGTCAGG-3′ and reverse 5′-AGTGAGGAACAAGCCAGAGC-3′ for *IL6*, and forward 5′-CATACTCCTGCTTGCTGATCC-3′ and reverse 5′-GATGCAGAAGGAGATCACTGC-3′ for *ACTB*.

### Western blot analysis

Primary culture of SMCs was treated with 100 ng/ml recombinant PDGF-BB (R&D Systems) or 100 ng/ml AREG (R&D Systems) for 72 h. Whole cell lysate was then prepared by a RIPA buffer (Sigma Aldrich, St. Louis, MO) supplemented with proteinase inhibitors and phosphatase inhibitors (Roche). Protein concentration was determined by a bicinchoninic acid (BCA) method (Pierce BCA Protein Assay Kit, Thermo Scientific). After Sodium Dodecyl Sulfate-Polyacrylamide gel electrophoresis (SDS-PAGE), separated proteins were transferred to a PVDF membrane (Hybond-P, GE healthcare, Buckinghamshire, UK) and blocked with an ECL plus blocking agent (GE healthcare). Membranes were then incubated with primary antibodies followed by incubation with anti-IgG antibody conjugated with horseradish peroxidase (GE healthcare). Finally, the signal was detected by a chemiluminescent reagent (ECL Prime Western Blotting Detection System, GE healthcare). α-Tubulin was served as an internal control.

The following primary antibodies were used: mouse monoclonal anti-SMA antibody (#M0851, Dako) and mouse monoclonal anti-α-tubulin antibody (#T6199, Sigma Aldrich).

### Statistical analysis

The power calculation for sample size estimation in each experiment was done by JMP Pro 14.0.0 software (SAS Institute, Cary, NC) using the data set obtained from the preliminary analyses or experiments. Data are shown as the mean ± SEM, and 2 groups were statistically compared using a Mann−Whitney U test. Statistical comparisons among more than 2 groups were conducted using a Kruskal−Wallis test followed by the Dunn’s test. A p value smaller than 0.05 was defined as statistically significant.

### Ethics approval and consent to participate

All of the following experiments including animal care and use complied with the National Institutes of Health Guide for the Care and Use of Laboratory Animals and the Animal Research Reporting *In Vivo* Experiments (ARRIVE) guidelines and were approved by the Institutional Animal Care and Use Committee of National Cerebral and Cardiovascular Center (approval number #17085, #18010, #19036) and of Kyoto University (#19008).

## Results

### Presence of dedifferentiated SMCs at the neck portion of IAs with the intimal hyperplasia in a rat model

**Figure 1:**
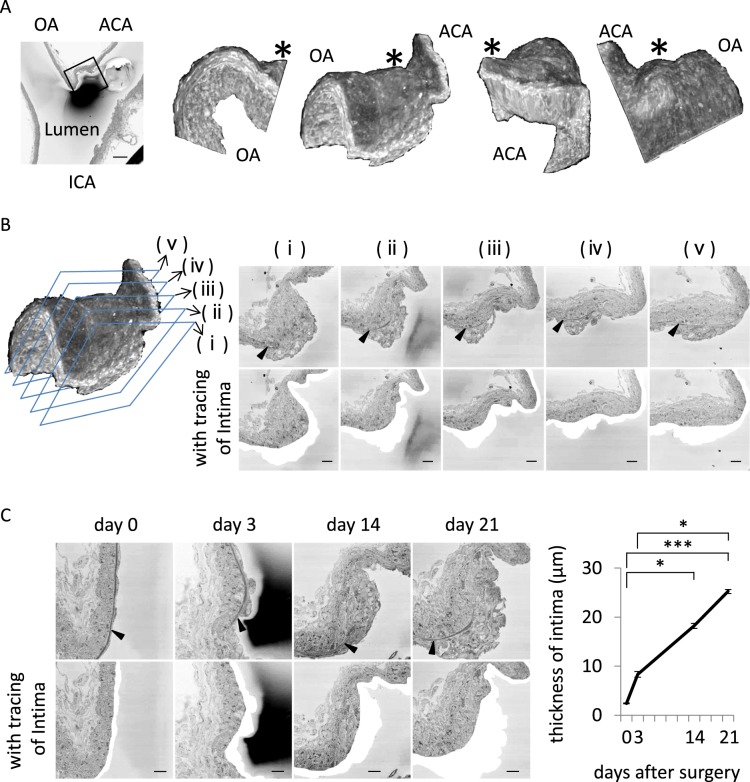
The formation of the intimal hyperplasia in intracranial aneurysm lesions. (**A**) The three-dimensional reconstruction of serial electron microscopic images acquired by serial block-face scanning electron microscopy (SBF-SEM) from the intracranial aneurysm (IA) lesion. On the 21^st^ day after IA induction, the IA lesions at the right anterior cerebral (ACA)-olfactory artery (OA) bifurcation were harvested and subjected to the analysis. The representative rotational images of the three-dimensional reconstructed microscopic images of the IA lesion corresponding to the square in the left panel are shown. Asterisk indicates the dome of the IA. Bar, 100 µm. (**B**) The formation of the intimal hyperplasia in IA lesions. Images labelled (i)-(v) show horizontal sections subjected to the SBF-SEM analysis. In the lower panels, the intima lied beneath the internal elastic lamina (IEL). Arrow heads indicate the IEL. Bars, 10 µm. (**C**) Increase in the size of the intimal hyperplasia of IA lesions during the progression of the disease. IA lesions induced at the right ACA-OA bifurcation of rat was harvested on the 3^rd^, 14^th^ or 21^st^ days after IA induction and subjected to the SBF-SEM analysis. In the lower panel, the intima lied beneath the IEL. Arrow heads indicate the IEL. Bars, 10 µm. The thickness of the thickest part in the intima was measured and shown in the right graph (n = 6). Statistical analysis was done by a Kruskal–Wallis test followed by the Dunn’s test. *p < 0.05. ***p < 0.001.

**Figure 2:**
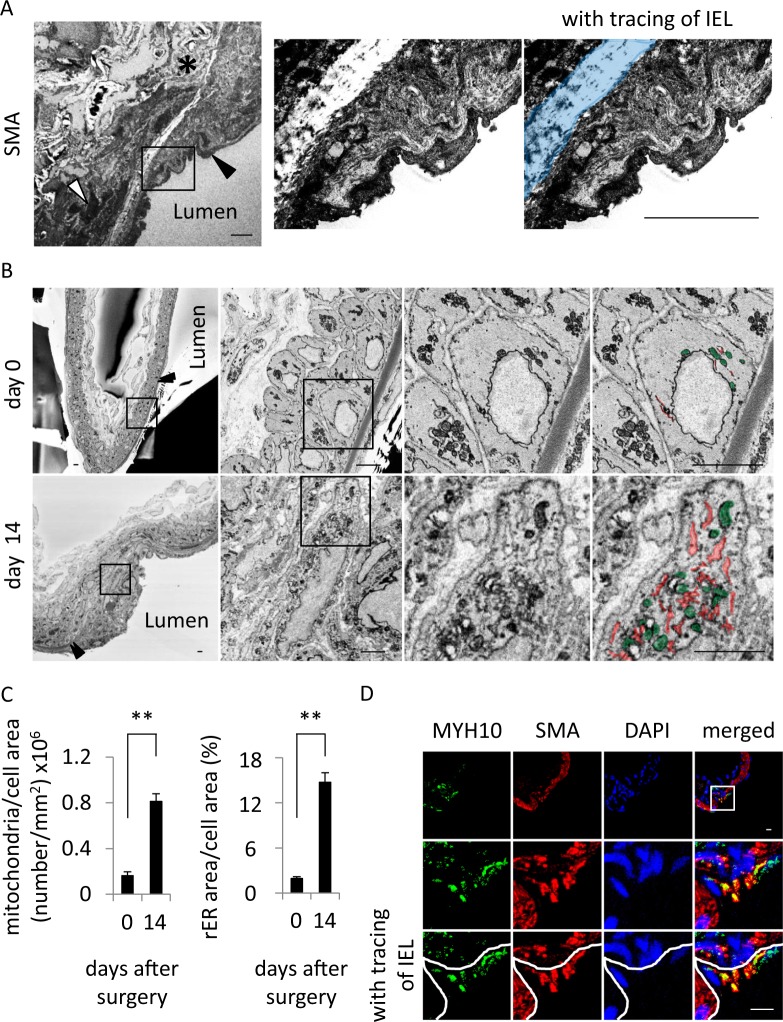
The presence of dedifferentiated smooth muscle cells in the intimal hyperplasia of intracranial aneurysm lesions. (**A**) The presence of smooth muscle α-actin (SMA)-positive smooth muscle cells (SMCs) in the intimal hyperplasia. On the 14^th^ day after intracranial aneurysm (IA) induction, the IA lesions at the right anterior cerebral-olfactory bifurcation were harvested and subjected to the immunoelectron microscopic analysis for a SMC marker, SMA. The black or white arrowhead indicates the cell positive for SMA staining in immunoelectron microscopic analysis in the intima or the media of the lesion, respectively. The asterisk indicates the cell negative for SMA staining. In the middle and right panels, the representative magnified images corresponding to the square in the left panel are shown and the internal elastic lamina (IEL) is colored blue in the right panel. Bars, 10 µm. (**B**,**C)** Increase of mitochondria and rough endoplasmic reticulum (rER) in SMCs present in the intimal hyperplasia of IA lesions. Mitochondria and rER were identified in control arterial walls and IA lesions at 14^th^ days after induction by scanning electron microscopic observation and the representative images are shown (**B**). The magnified images corresponding to the square in the left panels are shown in the right panels. Green or red color indicates mitochondria or rER, respectively. Arrow heads indicate the IEL. Bars, 2.5 µm. The number of mitochondria per unit cell area and the ratio of rER-present area over whole cell area in SMCs are shown in **c**. (**C**). Data represents the mean ± SEM (n = 6). Statistical analysis was done by a Mann–Whitney U test. **p < 0.01. (**D**) Expression of MYH10 in SMCs located at the intimal hyperplasia. IA lesions at 21 days after induction were harvested and subjected to the immunohistochemical analyses. The representative images of immunohistochemistry for a dedifferentiated SMC marker, MYH10, (green), SMA, (red), nuclear staining by DAPI (blue) and merged images are shown. In the lower panels, the representative magnified images corresponding to the square are shown. The IEL is traced in white. Bars, 10 µm.

To examine histopathological changes in IA walls during the progression of the disease, we used a rat model of IAs and subjected IA specimens to histopathological analyses. Intriguingly, we found that, not restrictedly but mainly at the neck portion of IAs, the intima locating beneath the internal elastic lamina (IEL) in the IA model was thicker (Fig. 1A,B) than that in control rats where such an intimal hyperplasia was absent (Figure S2). The thickness of the intimal hyperplasia gradually and significantly increased during the progression of the disease (Median; 2.4 µm (day 0), 8.7 µm (day 3), 18.0 µm (day 14), 24.8 µm (day 21)) (Fig. 1C). We then analyzed the cell component constituting the intimal hyperplasia present in IA lesions by immunoelectron microscopy. SMA-immunopositive SMCs were observed in the intima locating on the luminal side of IEL (Fig. 2A). Macrophages and endothelial cells were immunonegative for SMA, and SMA-immunopositive SMCs in tunica media were not stained in immunocontrol samples without 1st antibody (Figure S3). In the intimal hyperplasia, the accumulation of SMA-positive cells was observed and these cells consisted of a majority (Fig. 2A). Here, almost all SMCs in the intimal hyperplasia contained a large amount of mitochondria or rER and its number was significantly larger than that in SMCs present in the media of intracranial arteries (Median; 0.2 × 10^6^ (day 0), 0.8 × 10^6^ (day 14) in Fig. 2C left panel; 2.1% (day 0), 14.7% (day 14) in Fig. 2C right panel) (Fig. 2B,C, Figure S4), suggesting the activation of SMCs at the intimal hyperplasia. Many SMCs at the neck portion of IA lesions or some of SMCs at the dome also contained a large amount of mitochondria or rER (Figure S4). Because most SMCs at the intimal hyperplasia were positive for Myosin-10 (MYH10 also known as SMemb) in immunohistochemistry (Median; 0 µm^2^ (day 0), 6.2 µm^2^ (dome, day 21), 89.6 µm^2^ (neck, day 21) in Figure S5A left panel; 0 (day 0), 0.5 × 10^4^ (dome, day 21), 2.7 × 10^4^ (neck, day 21) in Figure S5A right panel; 0 µm^2^ (day 0), 4.1 µm^2^ (dome, day 21), 79.1 µm^2^ (neck, day 21) in Figure S5B left panel; 0 (day 0), 0.5 × 10^4^ (dome, day 21), 2.5 × 10^4^ (neck, day 21) in Figure S5B right panel) (Fig. 2D, Figure S5) and MYH10 is recognized as a marker for dedifferentiated SMCs^17,18^, dedifferentiated SMCs accumulate in the intimal hyperplasia. In case of media, most SMCs were negative for MYH10 staining (Fig. 2D) consistently with the result in electron-microscopic observation (Figure S4). Here, in female rats, the similar accumulation of dedifferentiated SMCs could be observed (Median; 1.9 µm (day 0), 23.5 µm (day 21) in Figure S6B) (Figure S6), confirming the absence of sex difference in this pathology.

### Potential of dedifferentiated SMCs as a regulator of chronic inflammation in the lesions

**Figure 3:**
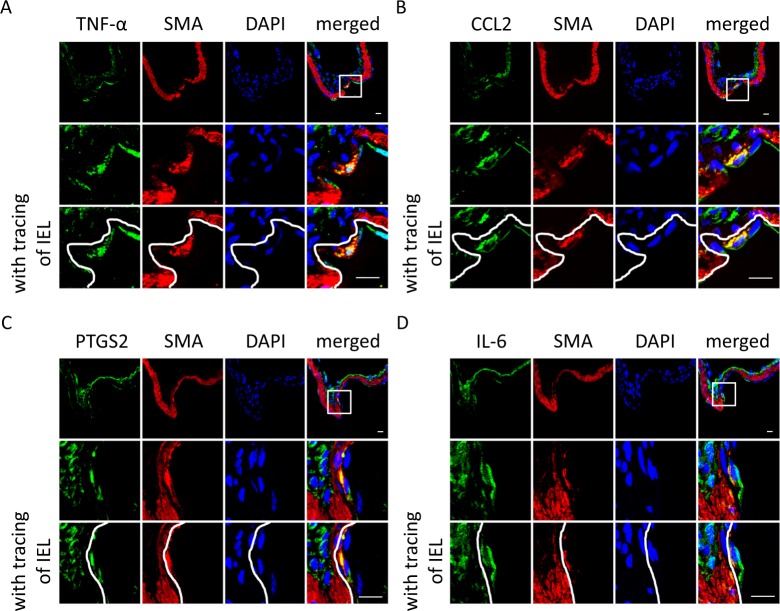
Expression of pro-inflammatory factors in dedifferentiated smooth muscle cells. On the 21^st^ day after intracranial aneurysm (IA) induction, the IA lesions at the right anterior cerebral-olfactory artery bifurcation were harvested and subjected to the immunohistochemical analyses. The representative images of immunohistochemistry for TNF-α (**A**), CCL2 (MCP-1) (**B**), PTGS2 (COX-2) (**C**) or IL-6 (**D**) (green), smooth muscle α-actin (SMA), a marker for smooth muscle cells, (red), nuclear staining by DAPI (blue) and merged images are shown. The internal elastic lamina (IEL) is traced in white. Bars, 10 µm.

We then examined expressions of pro-inflammatory factors, TNF-α [3, 56, 60], CCL2 (MCP-1)^19,20^, PTGS2 (COX-2)^8,21^ and IL-6^22,23^ as a marker of chronic inflammatory responses in lesions, in immunohistochemistry, because chronic inflammation plays a pivotal role in the pathogenesis^8,12,24–26^. Expression of all these pro-inflammatory factors was detected at the adventitia and also at the intimal hyperplasia lied beneath the disrupted IEL (Fig. 3, Figure S7). As previously reported, expression of these cytokines at the adventitia is mainly from macrophages and fibroblasts^8,14,19,27^. Cells positive for TNF-α, CCL2, PTGS2 or IL-6 staining at the intimal hyperplasia were also positive for SMA (Fig. 3). This observation thus means that dedifferentiated SMCs locating at the intimal hyperplasia produced pro-inflammatory factors *in situ*. Here, intriguingly, most SMCs at the media, where most SMCs were not dedifferentiated (Figure S4), did not produce TNF-α, CCL2, PTGS2 or IL-6 (Fig. 3). Because some SMCs present in the intimal hyperplasia expressed CD68 in immunohistochemistry (Figure S8A) and also contained lipid depositions and became foam cells (Figure S8B), dedifferentiated SMCs presumably play a pivotal role in the pathogenesis of IAs via regulating inflammatory responses as in atherosclerosis^28,29^. Thereby, the recruitment to the intimal hyperplasia and the dedifferentiation is presumably crucial step for SMCs to evoke and maintain inflammation *in situ*.

### Potential factors regulating migration of SMCs to form the intimal hyperplasia in IA lesions

In a nod to potential contribution of dedifferentiation of SMCs and migration of these cells in the intimal hyperplasia to form inflammatory microenvironment leading to the progression of the disease, we next aimed to identify factors regulating dedifferentiation and migration of SMCs to form the intimal hyperplasia in IA lesions.

**Figure 4:**
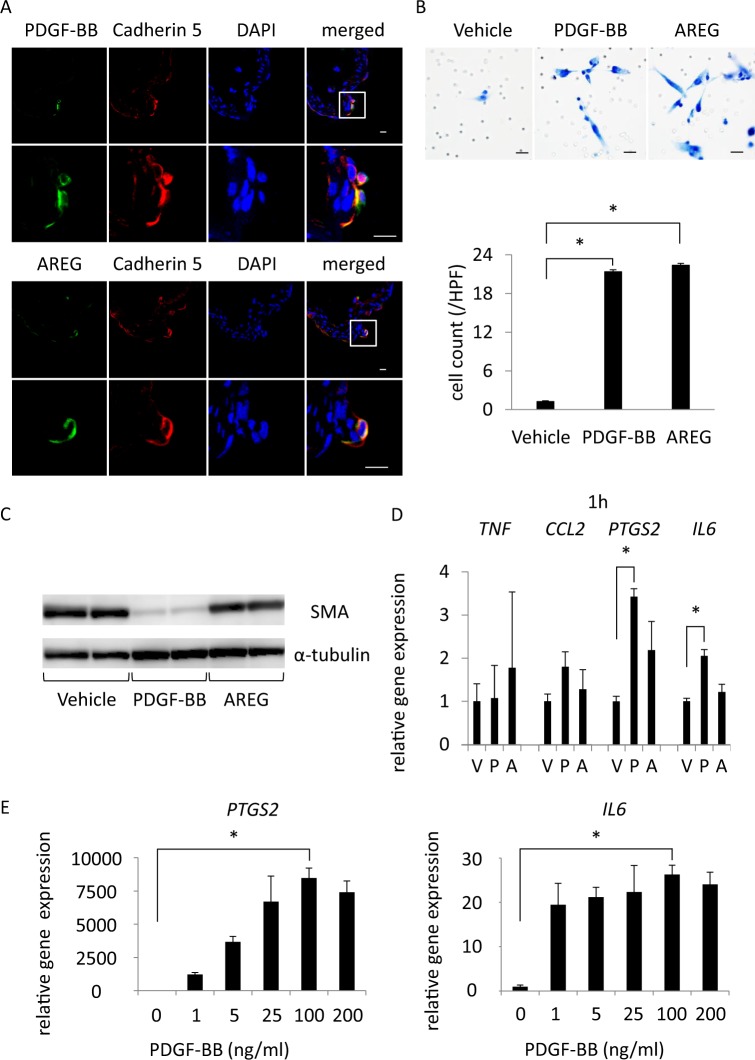
PDGF-BB as a factor for smooth muscle cells to migrate, dedifferentiate and express pro-inflammatory genes. (**A**) Expression of PDGF-BB or Amphiregulin (AREG) in endothelial cells of intracranial aneurysm (IA) lesions. On the 14^th^ day after IA induction, IA lesions at the right anterior cerebral-olfactory artery bifurcation were harvested and subjected to the immunohistochemical analyses. The representative images of immunohistochemistry for PDGF-BB or AREG (green), Cadherin 5, a marker for endothelial cells, (red), nuclear staining by DAPI (blue) and merged images are shown. The magnified images corresponding to the square in the upper panels are shown in the lower panels. Bars, 10 µm. (**B**) Chemotactic activity of PDGF-BB or AREG on smooth muscle cells (SMCs). The migration of primary culture of SMCs across matrigel-coated pores via 100 ng/ml PGDF-BB or 100 ng/ml AREG was assessed by a transwell system. The representative images of SMCs migrated are shown. Bars, 10 µm. The number of migrated cells is shown in the lower graph. Data represents the mean ± SEM (n = 4). Statistical analysis was done by a Kruskal–Wallis test. *p < 0.05. (**C**) Dedifferentiation of SMCs by PDGF-BB. Primary culture of SMCs were stimulated with 100 ng/ml PGDF-BB or 100 ng/ml AREG for 72 h and expression of SMA was assessed by western blot analysis using α-tubulin as an internal control. The representative images are shown. (**D,E**) Induction of *PTGS2* (COX-2) or *IL6* by PDGF-BB in cultured SMCs. Primary culture of SMCs were stimulated with vehicle (V), 100 ng/ml PGDF-BB (P) or 100 ng/ml AREG (**A**) (**D**) or each dose of PDGF-BB (**E**) for 1 h and expression of *TNF*, *CCL2*, *PTGS2* or *IL6* was examined by quantitative RT-PCR analysis. Data represents the mean ± SEM (n = 4). Statistical analysis was done by a Kruskal–Wallis test. *p < 0.05.

We first examined a mechanism regulating the migration of SMCs using a primary culture of SMCs from human carotid artery. We selected PDGF-AA, PDGF-BB, NFE2L2 (Nrf-2) and AREG as potential factors responsible for the migration of SMCs into the intimal hyperplasia, referencing the previous reports^30–35^. In immunohistochemistry, PDGF-BB expression was detectable at the Cadherin 5-positive endothelial cells mainly at the neck portion of the lesions (Fig. 4A) but the expression was not observed in endothelial cells located at the bifurcation site without IA induction (Figure S9). AREG was also expressed in endothelial cells restrictedly of the lesions (Fig. 4A, Figure S9). Both factors were thus induced in endothelial cells of the lesion during the progression of the disease (Median; 0 µm^2^ (day 0), 1.1 µm^2^ (dome, day 14), 66.9 µm^2^ (neck, day 14) in the left panel; 0 (day 0), 0.2 × 10^4^ (dome, day 14), 1.2 × 10^4^ (neck, day 14) in the right panel) (Figure S10). In contrast, PDGF-AA was expressed in endothelial cells ubiquitously of intracranial arterial walls, not restrictedly of IA lesions (Figure S11). NFE2L2 was only partially activated and accumulated in nuclei of MYH10-positive dedifferentiated SMCs, suggesting the limited contribution of Nrf-2 activation to the inhibition of dedifferentiation of SMCs in the intimal hyperplasia (Figure S12). To corroborate whether PDGF-BB and AREG could indeed facilitate the migration of SMCs to form the intimal hyperplasia at the neck portion of the lesions, we examined effect of these factors on the migration of primary culture of SMCs in a transwell system. Addition of PDGF-BB or AREG in a lower chamber both significantly facilitated migration of cultured SMCs across matrigel-coated pores (Fig. 4B).

We next examined whether PDGF-BB or AREG could induce the dedifferentiation of SMCs as observed in IA lesions (Fig. 2D). Addition of PDGF-BB not AREG in a culture medium remarkably reduced protein expression of SMA, a SMC marker, in primary culture of SMCs (Fig. 4C, Figure S13), providing the *in vitro* evidence supporting the induction of the dedifferentiation of SMCs by PDGF-BB. As well known that dedifferentiated SMCs produce various pro-inflammatory factors^17,28,36^, the dedifferentiated SMCs in IA lesions produced pro-inflammatory factors like TNF-α (Fig. 3). We therefore examined effect of PDGF-BB or AREG on expression of pro-inflammatory genes in primary culture of SMCs. As a result, PDGF-BB not AREG induced expression of *PTGS2*, a gene encoding COX-2, and *IL6* in cultured SMCs at a time- and concentration-dependent manner (Fig. 4D,E, Figure S14).

These results combined together suggest that PDGF-BB induced in endothelial cells of IA lesions facilitates the dedifferentiation, the migration and also the induction of some pro-inflammatory factors and contributes to the formation of inflammatory microenvironment in the intimal hyperplasia leading to the progression of IAs.

### Induction of PDGF-BB in endothelial cells loaded on high wall shear stress (WSS)

**Figure 5:**
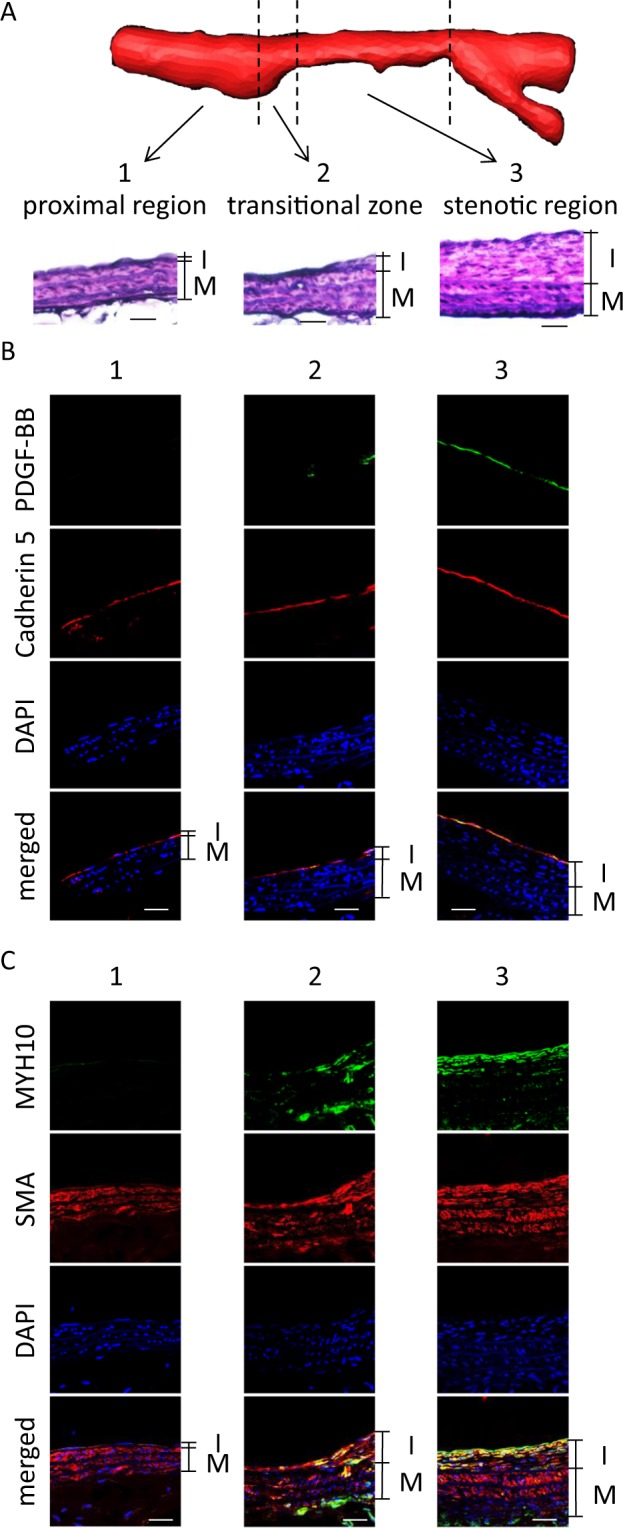
Induction of PDGF-BB and MYH10 in the transitional and the stenotic regions. (**A**) The intimal hyperplasia induced in a stenosis model. The three-dimensional reconstructed image of MRI of a stenosis model and images of elastic van gieson staining (EvG) corresponding to the proximal, the transitional or the stenotic region are shown. I or M indicates the intima or the media, respectively. Bars, 20 µm. (**B**,**C**) Induction of PDGF-BB or MYH10 in the transitional and the stenotic region of a stenosis model. On the 30^th^ day after surgical manipulations to induce stenosis at the left common carotid artery (CCA), the left CCA were harvested and subjected to the immunohistochemical analyses. The representative images of immunohistochemistry for PDGF-BB (green in **B**), MYH10 (green in **C**), Cadherin 5, a marker for endothelial cells, (red in **B**), smooth muscle α-actin (SMA), a marker for smooth muscle cells, (red in **C**), nuclear staining by DAPI (blue) and merged images are shown. 1, 2 or 3 indicates the proximal region, the transitional zone or the stenotic region, respectively. Bars, 20 µm.

We hypothesized that high WSS induced PDGF-BB expression to recruit SMCs at the neck portion of IAs to form the intimal hyperplasia referencing previous reports demonstrating the crucial role of high WSS in expression of this protein in cultured endothelial cells^37^. Also high WSS is loaded on endothelial cells at the neck portion of IA lesions by a computed fluid dynamics analyses^12,13,38,39^. To acquire an *in vivo* evidence that high WSS induces PDGF-BB expression in endothelial cells, we used a stenosis model of a carotid artery in a rat^40,41^ and examined expressions of PDGF-BB in the lesions. In this model, the region loaded on high WSS can be observed mainly at the transitional or stenotic region^40,42^ because WSS is inversely proportional to radius of arteries. Consistent with the above hypothesis, in the stenosis model of a carotid artery, the expression of PDGF-BB was induced in Cadherin 5-positive endothelial cells specifically at the transitional or the stenotic region where high WSS was loaded (Fig. 5A,B, Figures S15 and S16). Note that the intimal hyperplasia occurred at the region positive for PDGF-BB staining in immunohistochemistry and MYH10-positive dedifferentiated SMCs accumulated there (Fig. 5C, Figures S15 and S16). These findings supported our assumption that high WSS induced PDGF-BB expression to recruit SMCs to form the intimal hyperplasia.

### The loss and the morphological changes of medial SMCs during the progression of IAs

The degenerative change of medial SMCs including the loss of them is one of the well-recognized histopathological features of IA lesions^1,3,5^. Consistently, in our observation, we found the remarkable loss in number and the significant change in morphology in medial SMCs (Figure S17). The number of medial SMCs was gradually and significantly reduced according to the disease progression after IA induction in a rat model (Median; 7,000 (day 0), 4,400 (day 3), 3,600 (day 7), 2,600 (day 14), 1,200 (day 21) in Figure S17B) (Figure S17). Morphologically, in the three-dimensional reconstructed electron microscopic examination of IA lesions at each step of the disease progression, medial SMCs underwent the remarkable morphological changes (Figs S18A,B). Until the 21^st^ day after IA induction, they reduced their volume but did not significantly change the surface area (Median; 2,060 µm^3^ (day 0), 1,413 µm^3^ (day 21) in the left panel; 1,802 µm^2^ (day 0), 1,702 µm^2^ (day 21) in the right panel) (Figure S18C), suggesting the flattering of the cell. Consistently, at the cross section of medial SMCs after the three-dimensional reconstruction, the ratio of the short axis over the long axis was significantly smaller than that in medial SMCs from normal intracranial arterial wall (Median; 4.3 µm (day 0), 2.0 µm (day 21) in the left panel; 8.0 µm (day 0), 17.8 µm (day 21) in the middle panel; 0.5 (day 0), 0.1 (day 21) in the right panel) (Figure S18D).

## Discussion

One of the major limitations regarding the present study is the lack of the experimental evidence from human specimens partially because, in microsurgery to treat IAs, only a tiny specimen only from the dome, not the neck portion, of the lesion can be harvested. Until today, others have reported the presence of the intimal hyperplasia^43^ and also the presence of MYH10-positive dedifferentiated SMCs in ruptured IA lesions^44^. Also another pathological study demonstrating the presence of S100A4-positive dedifferentiated SMCs and the marked decrease of SMA expression in human lesions^1^ may support the contribution of dedifferentiated SMCs in the pathogenesis. Because in human studies we cannot get adequate information especially about the time course of the disease progression, the findings using a rat model will compensate such a limitation. Another major limitation is the lack of *in vivo* evidence about a factor mediating dedifferentiation and/or recruitment of SMCs although we indicated the contribution of PDGF-BB produced by endothelial cells in lesions. Importantly, the crucial role of PDGF signaling in the pathogenesis of atherosclerosis has been revealed^45^. In this study, the gain-of-function knock-in in PDGFRβ locus (PDGFRβ^D849V^) in mice results in the exacerbation of atherosclerosis including the increase of inflammatory cell migration, the promotion of the proliferation of SMCs and the facilitation of the plaque formation^45^. Previous studies above and the review article^4^ thus support our hypothesis. In addition, our observation that PDGF-BB expression is induced in endothelial cells where high WSS is loaded is consistent with the previous *in vitro* studies that shear stress-loading increases expression of PDGF-BB in cultured endothelial cells^46,47^. Chemotactic activity of PDGF-BB for SMCs is also supported by the previous studies^31,46^. If a rat line deficient in *Pdgfb* which encodes PDGF-BB is available, we can actually examine more accurate effect of PDGF-BB on the pathogenesis. However, unfortunately, the mouse line deficient in this gene is a lethal because of the loss of pericytes and resultant fatal hemorrhage during development^48,49^. A rat line deficient in *Pdgfb* specifically in endothelial cells is therefore necessary. But, unlike in mice, the establishment of such a line in rats is challenging because we need to establish at least two lines; the line flanked *Pdgfb* and the endothelial cell-specific Cre-recombinase expressing line. Alternatively, the inhibition of this protein by a specific antibody may be useful to further accurately validate the role of this protein in the pathogenesis of IAs. We however failed to find out a monoclonal antibody to neutralize the action of PGDF-BB in rats. This study is thus limited as it provides no causal evidence, yet correlative findings suggesting PDGF a potential contributing factor.

Accumulation of experimental findings obtained from animal models of IAs and from histopathological analyses using human IA specimens has clarified the crucial role of long-lasting inflammation, so-called chronic inflammation, in the initiation and the progression of the disease^8,19,20,24–27,50,51^. In this process, the crucial contribution of macrophages to triggering and maintenance/exacerbation of inflammatory responses has been well highlighted^8,19,20,25,26^. Because only a few resident macrophages can be observed in intracranial arteries in the circle of Willis and intracranial arteries lack vasa vasorum in most of cases^52^, these macrophages infiltrates across endothelial cell barrier. In the process of the disease development, endothelial cells are activated by high WSS during the initiation of the disease and presumably by excessive low WSS and turbulent flow during the progression^12,53–55^ and actively participate in inflammatory responses in lesions, e.g. activation of NF-κB and expression of chemoattractants for macrophages to support their recruitment *in situ*^8,26^. Endothelial cells are damaged and lost during the progression of IAs. In this step, the integrity of endothelial cell barrier is affected^56^ and macrophage infiltration is greatly facilitated. However, the contribution of SMCs to inflammatory responses in microenvironment of the disease is not clear although they are a major type of cells in arterial walls. We in the present study clarified the accumulation of dedifferentiated SMCs in the intimal hyperplasia mainly at the neck portion of IA lesions and expression of pro-inflammatory factors related to the pathogenesis of IA like TNF-α^27,50,51^, CCL2 (MCP-1)^19,20^ or PTGS2 (COX-2)^8,21^ from these cells, suggesting the role of SMCs especially dedifferentiated ones to the formation of inflammatory microenvironment leading to the progression of the disease. The results from histopathological analyses enrolling human IA lesions that IA lesions with the intimal hyperplasia contained proliferating SMCs and inflammatory infiltrates including macrophages^43^ may indicate the clinical relevance of our study. In this process, a high WSS loaded on the neck of IA lesions may contribute to not only the recruitment but also the dedifferentiation of medial SMCs via PDGF-BB. In atherosclerosis, the formation of the intimal hyperplasia (plaque) and the accumulation of dedifferentiated SMCs there becomes the histopathological feature. Furthermore, because the lineage-tracing studies have clarified that the significant part of CD68-positive cells in plaque is derived from SMCs, dedifferentiated SMCs plays the key role to mediate inflammatory responses in lesions^17,29,57,58^. Dedifferentiated SMCs thereby play a crucial role in the pathogenesis of atherosclerosis. Considering the expression of pro-inflammatory factors in dedifferentiated SMCs accumulated in the intimal hyperplasia of IA lesions and also the expression of CD68 in these cells (Figure S8A), these SMCs may play an important role to the maintenance of inflammatory responses promoting the disease as well. Furthermore, SMCs could induce the phenotypic alternation in endothelial cells^59^ and may further contribute to the progression of the disease. The above assumption may be supported by the histopathological study using human IA specimens that SMA-positive foam cells was present in about half of IA lesions and the accumulation of oxidized lipids was correlated with degenerative changes in media^60^. If so, IA shares the similar machineries in the pathogenesis with atherosclerosis in terms of the contribution of dedifferentiated SMCs.

## Conclusions

SAH due to rupture of IAs is a lethal disease. Because SMC is a major cell component consisting intracranial arterial walls, understanding of a contribution of this type of cell to the pathogenesis of IAs is mandatory. In the present study, we have clarified the accumulation of the dedifferentiated SMCs in the intimal hyperplasia at the neck portion of IA lesions. The dedifferentiated SMCs express various pro-inflammatory factors to form the inflammatory microenvironment there leading to the progression of the disease. In this process, PDGF-BB is induced in endothelial cells loaded on high WSS and functions to facilitate the dedifferentiation and migration of SMCs to form the intimal hyperplasia. The findings from the present study highlight the potential of the dedifferentiated SMCs to mediate inflammatory responses leading to the progression of the disease and have successfully identified some therapeutic targets like PDGF-BB to prevent rupture of IAs.

## Supplementary information

Supplementary Information.

## Data Availability

The datasets used and/or analyzed during the current study available from the corresponding author on reasonable request.
