# Supplementary material for: Dedifferentiation of smooth muscle cells in intracranial aneurysms and its potential contribution to the pathogenesis
Source: Sci Rep. 2020 May 20;10:8330. doi: 10.1038/s41598-020-65361-x (PMC7239886; doi:10.1038/s41598-020-65361-x)
Supplement: Supplementary file 1 — Supplementary Information. [file 41598_2020_65361_MOESM1_ESM.pdf]

Dedifferentiation of smooth muscle cells in intracranial aneurysms and its potential contribution to the pathogenesis

Mieko Oka, Satoshi Shimo, Nobuhiko Ohno, Hirohiko Imai, Yu Abekura, Hirokazu Koseki, Haruka Miyata, Kampei Shimizu, Mika Kushamae, Isao Ono, Kazuhiko Nozaki, Akitsugu Kawashima, Takakazu Kawamata, Tomohiro Aoki

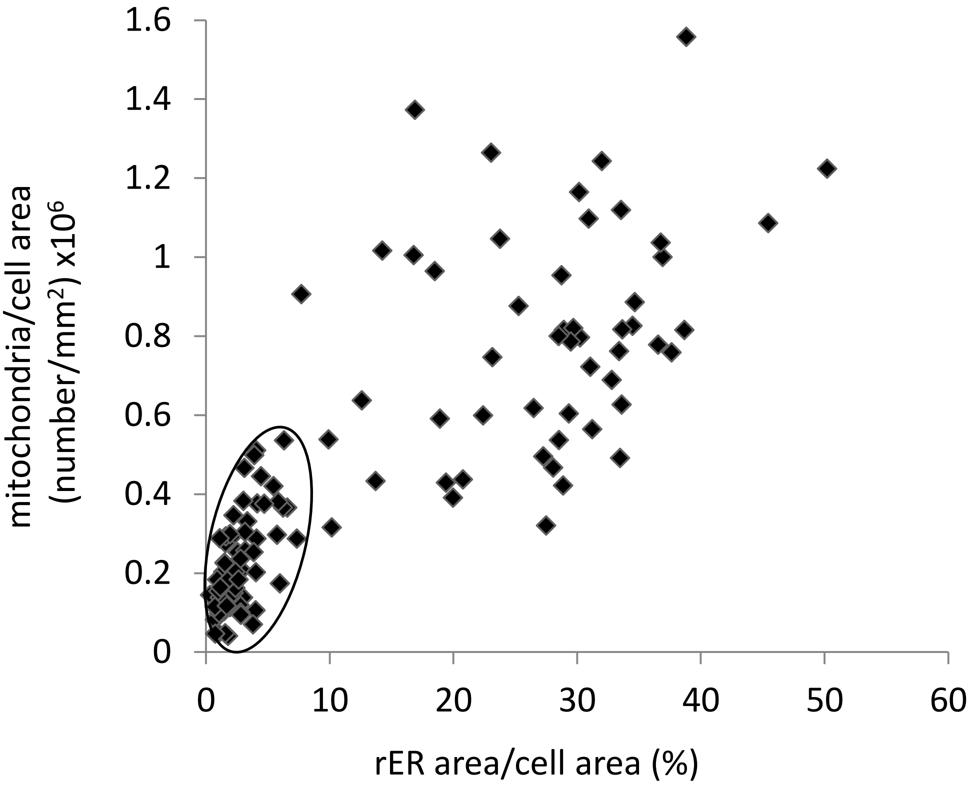

Figure S1. The quantification of mitochondria and rough endoplasmic reticulum in smooth muscle cells in intracranial aneurysms.

Scatter plots were created with the vertical axis representing the number of mitochondria and the horizontal axis representing the ratio of rough endoplasmic reticulum (rER) area over whole cell area. Cells with more than 0.6 x 10^6 /mm^2 mitochondria and more than 8 % rER area over whole cell area were considered as activated smooth muscle cells. The circle indicates non-activated cells.

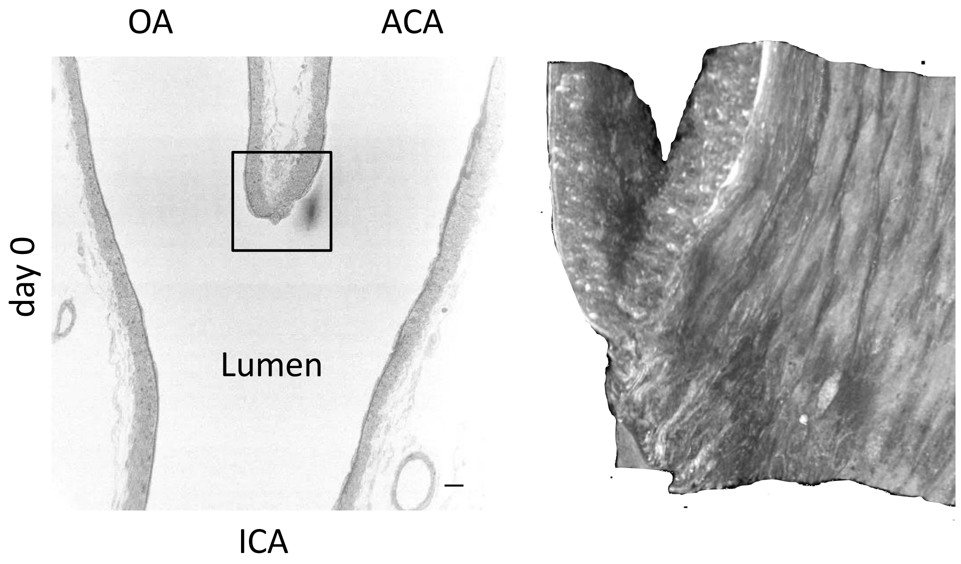

Figure S2. The three-dimensional reconstructed images of the bifurcation site before intracranial aneurysm induction.

The right anterior cerebral (ACA)-olfactory artery (OA) bifurcation before intracranial aneurysm induction (day 0) was harvested and subjected to the serial block-face scanning electron microscopic observation. The three-dimensional reconstructed image corresponding to the square in the left panel is shown in the right panel. ICA; internal carotid artery. Bar, 10 µm.

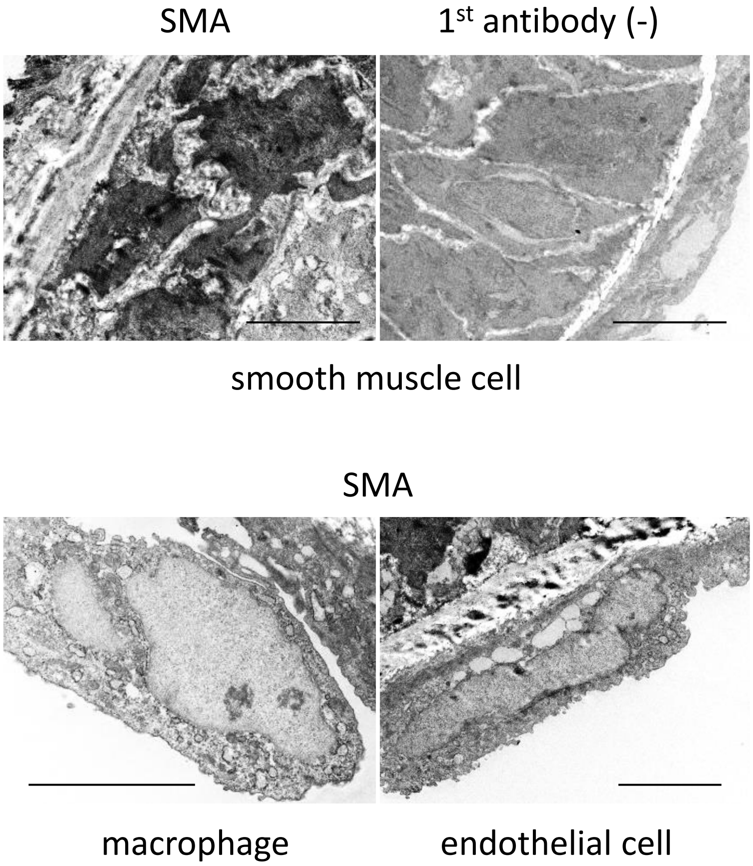

Figure S3. The specificity of the primary antibody used in immunoelectron microscopic examinations on smooth muscle cells.
On the 14th day after intracranial aneurysm (IA) induction, the IA lesions at the right anterior cerebral-olfactory artery bifurcation were harvested and subjected to immuno-electron microscopic examination for smooth muscle α-actin (SMA), a marker for smooth muscle cells (SMCs). The immunostaining without the primary antibody was done as a negative control study. Bars, 10 µm. Note the specificity of the primary antibody used on SMCs.

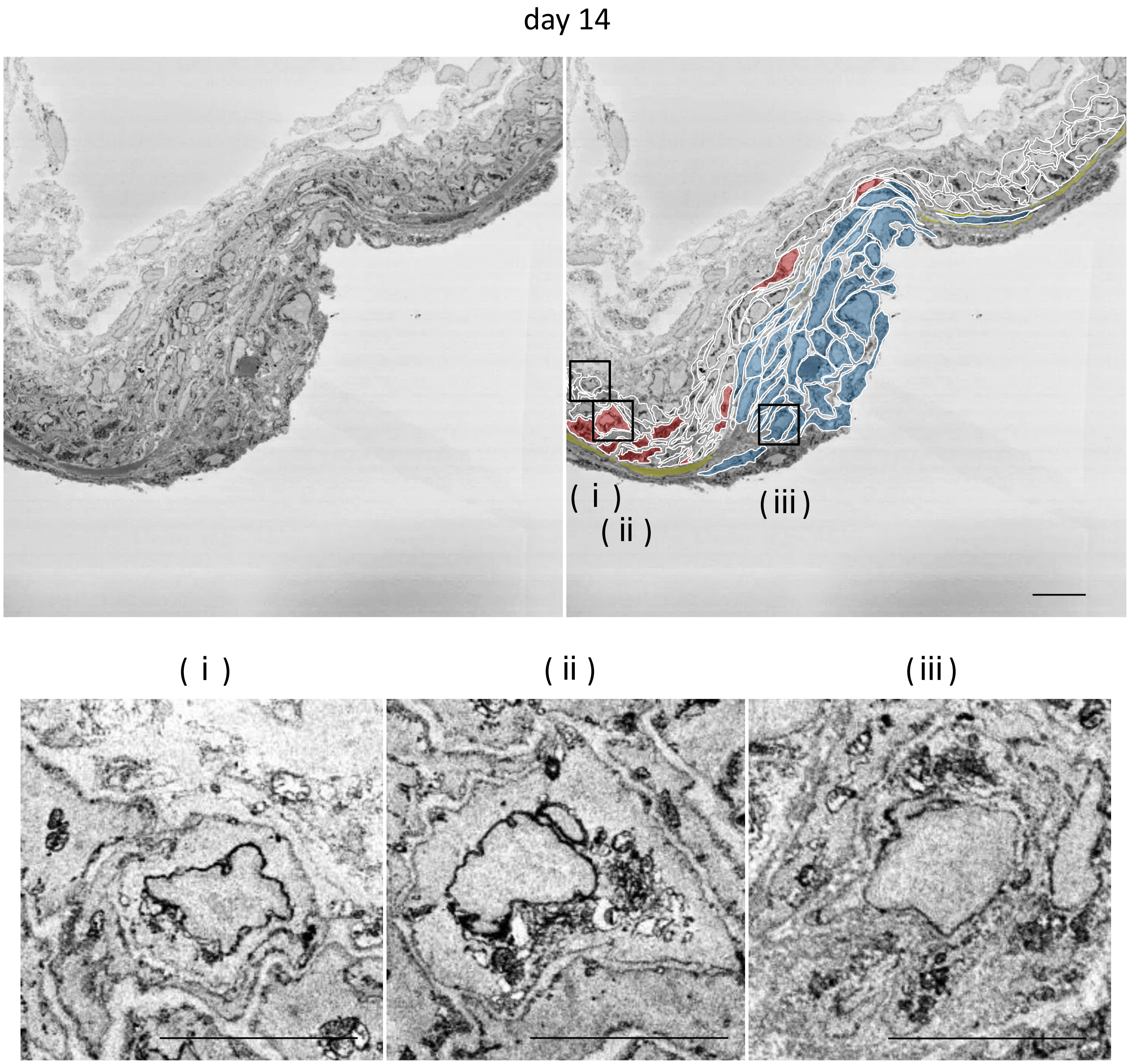

Figure S4. Distribution of dedifferentiated smooth muscle cells in intracranial aneurysm lesions. On the 14th day after intracranial aneurysm (IA) induction (day 14), the IA lesions at the right anterior cerebral-olfactory artery bifurcation were harvested and subjected to the serial block-face scanning electron microscopic observation. Each smooth muscle cell (SMC) is traced in white. Dedifferentiated SMCs in the media (red), ones in the intimal hyperplasia (blue) or the internal elastic lamina (yellow) are visualized. In the lower panels, the representative magnified images corresponding to the square are shown. Bars, 10 μm.

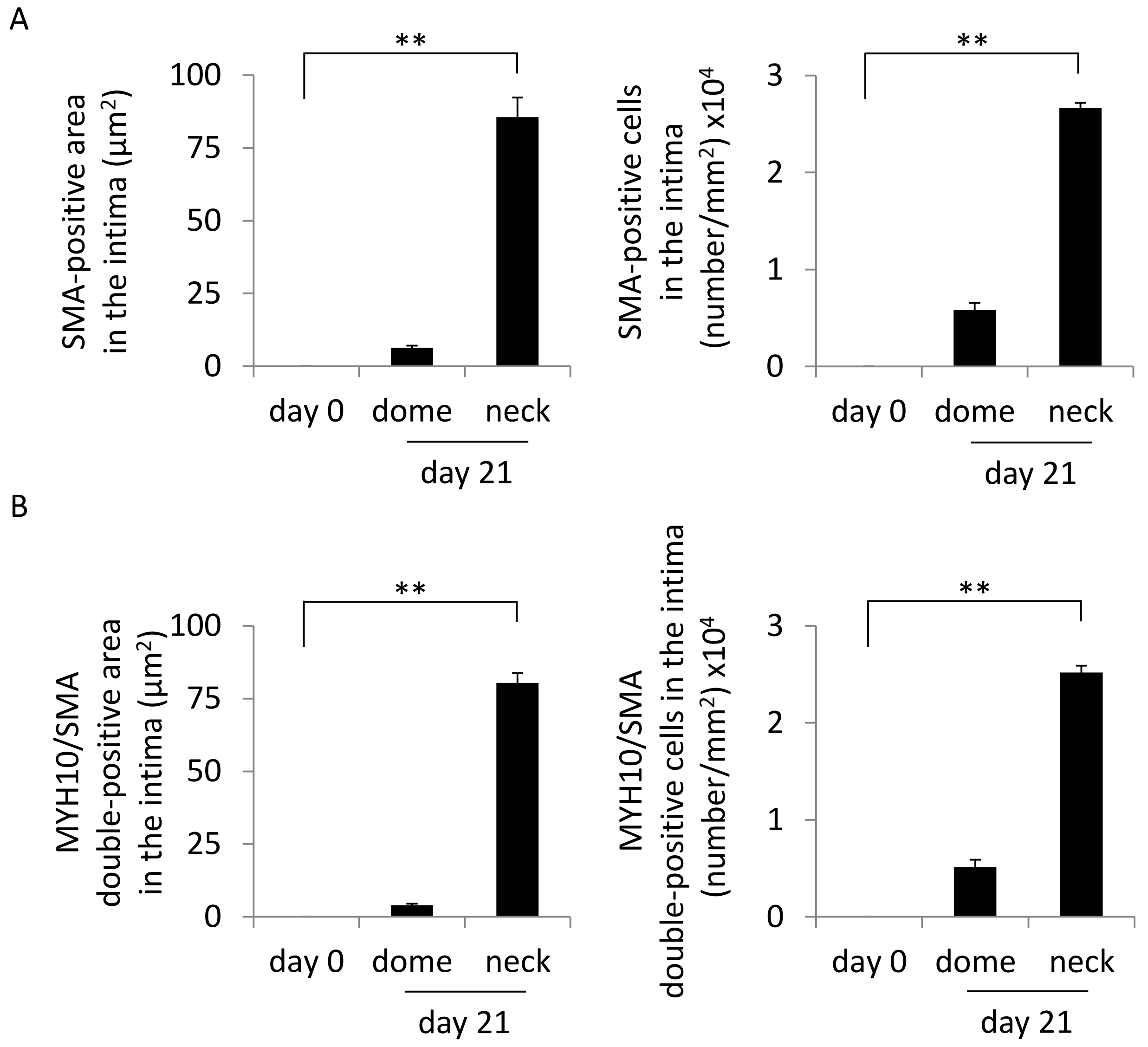

Figure S5. The quantified data of smooth muscle cells and dedifferentiated smooth muscle cells in the intimal hyperplasia of intracranial aneurysm lesions.

Before (day 0) or on the 21st day after intracranial aneurysm (IA) induction (day 21), IA lesions at the right anterior cerebral-olfactory artery bifurcation were harvested and subjected to the immunostaining for smooth muscle α-actin (SMA) and myosin heavy chain 10 (MYH10). The area of signals positive for SMA staining or double positive for SMA and MYH10 in immunohistochemistry in the intima and the number of SMA-positive cells or SMA/MYH10-double positive cells in the intima per the area of whole intracranial aneurysm lesions are shown in **A** or **B**, respectively. Data represents the mean ± SEM (n=5). Statistical analysis was done by a Kruskal–Wallis test followed by the Dunn's test. **; $p < 0.01$.

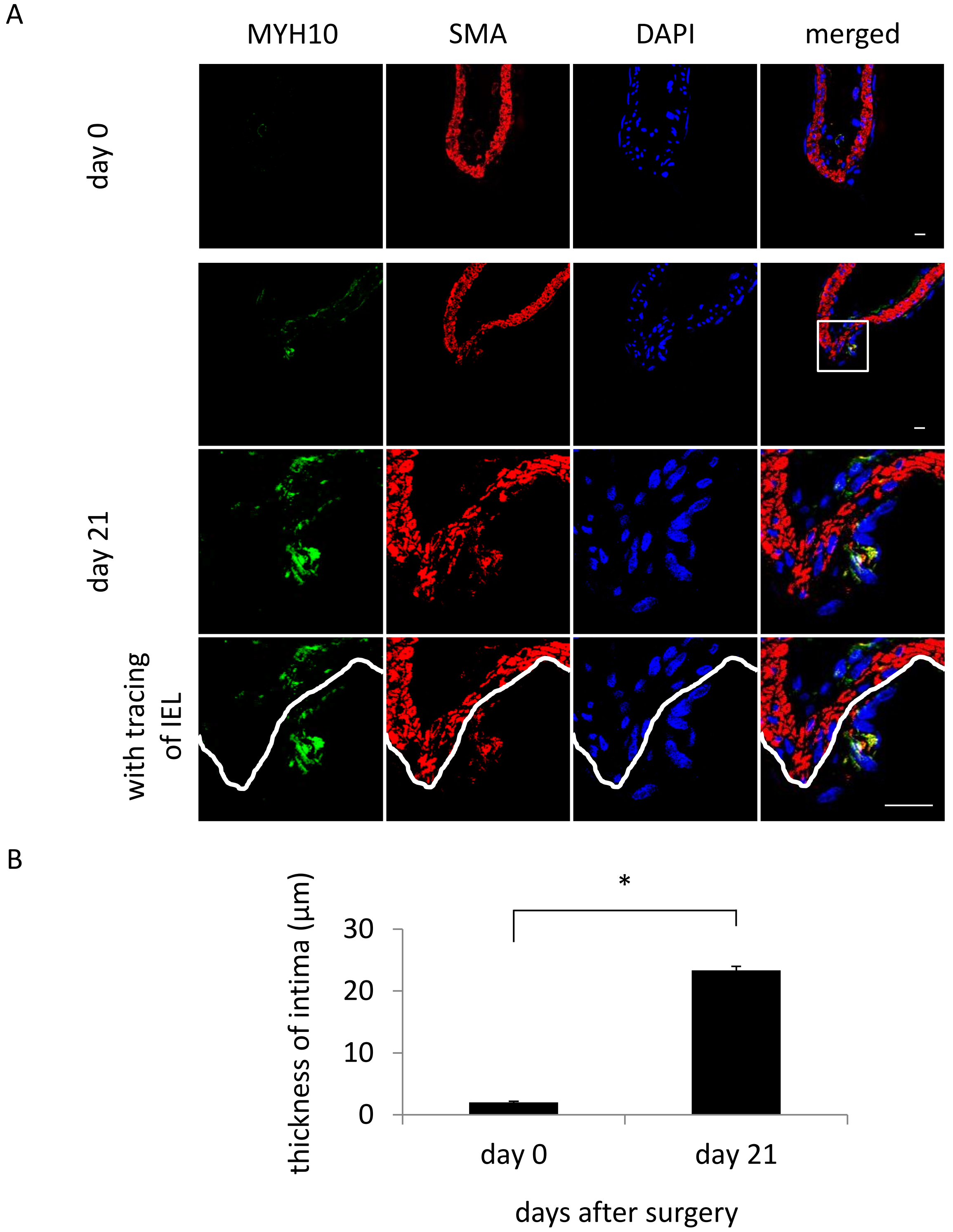

Figure S6. The presence of dedifferentiated smooth muscle cells in the intimal hyperplasia of intracranial aneurysm lesions induced in female rats.
A Expression of myosin heavy chain 10 (MYH10) in smooth muscle α-actin (SMA)-positive smooth muscle cells (SMCs) located in the intimal hyperplasia of female rats. Before (day 0) or on the 21st day after intracranial aneurysm (IA) induction (day 21), IA lesions at the right anterior cerebral-olfactory artery bifurcation were harvested and subjected to the immunostaining for SMA and MYH10. The representative images of immunohistochemistry for a dedifferentiation marker, MYH10 (green), SMA (red), nuclear staining by DAPI (blue) and merged images are shown. In the lower panels, the magnified images corresponding to the square are also shown. The internal elastic lamina (IEL) is traced in white. Bars, 10 μm. **B** Increase in the size of the intimal hyperplasia of IA lesions induced in female rats. The thickness of the thickest part in the intima was measured. Data represents the mean ± SEM (n=5). Statistical analysis was done by a Mann-Whitney U test. *; $p < 0.05$.

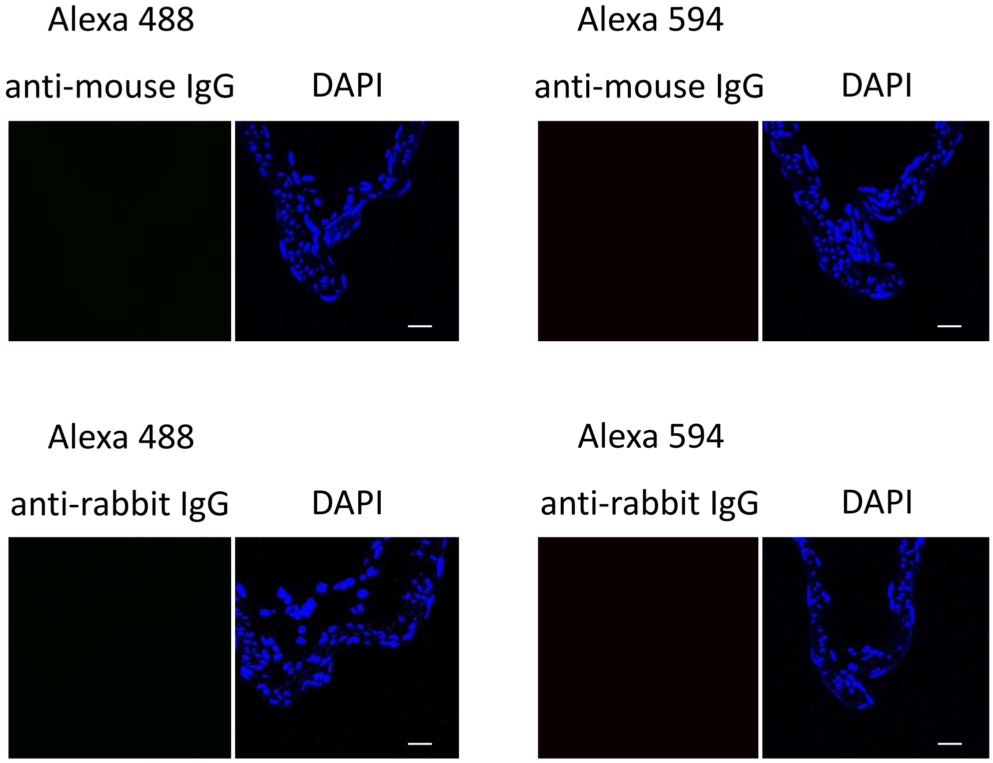

Figure S7. Immunostaining of intracranial aneurysm lesions without primary antibodies as a negative control study.

The representative images of immunohistochemistry of intracranial aneurysm lesions using only secondary antibodies, (Alexa 488-conjugated anti-mouse IgG, Alexa 488-conjugated anti-rabbit IgG, Alexa 594-conjugated anti-mouse IgG, Alexa 594-conjugated anti-rabbit IgG), are shown with nuclear staining by DAPI (blue). Bars, 10 μm.

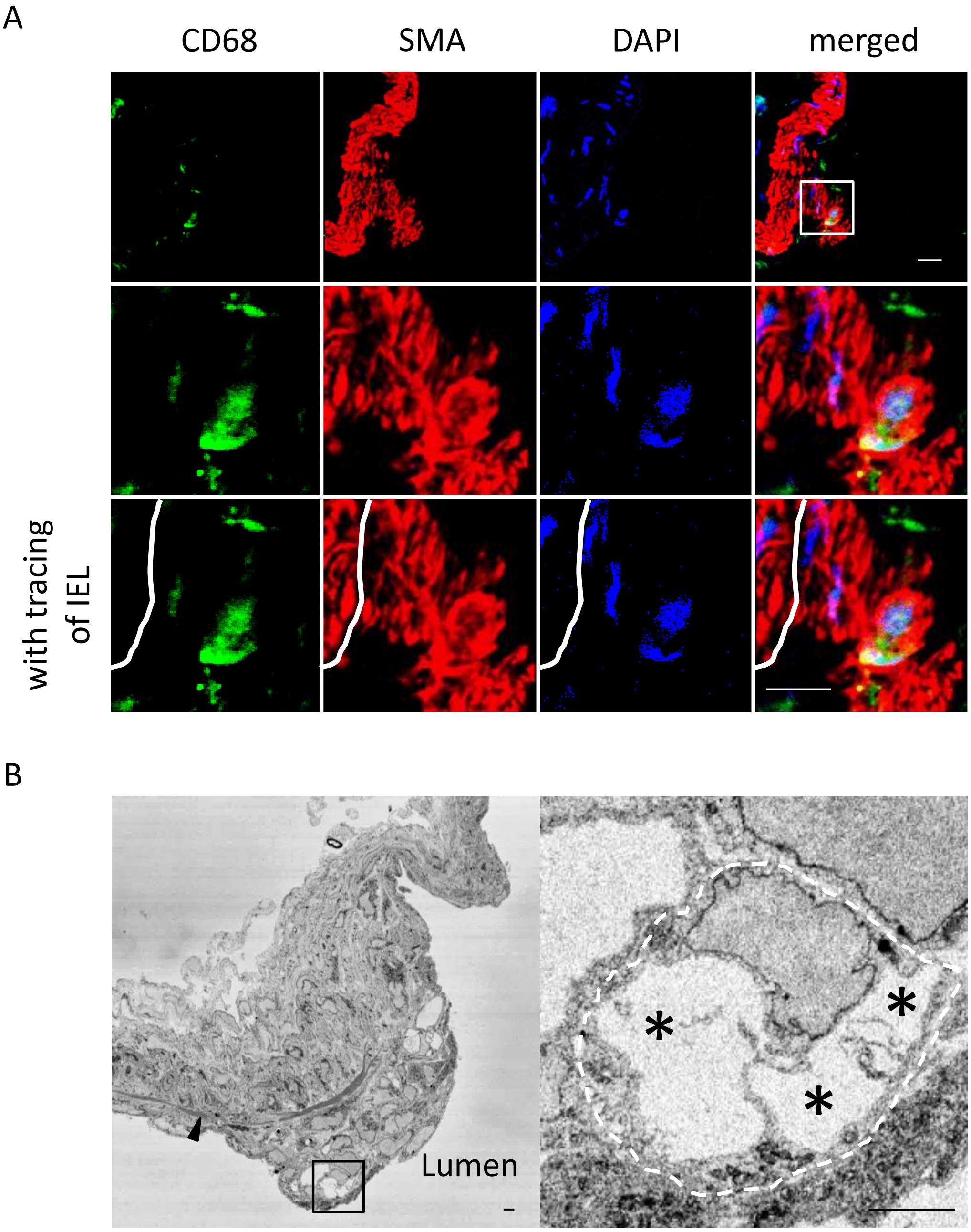

Figure S8. CD68-positive smooth muscle cells and foam cells in the intimal hyperplasia of intracranial aneurysm lesions.

A Expression of CD68 in smooth muscle cells (SMCs) in the intimal hyperplasia of intracranial aneurysm (IA) lesions. On the 14th day after IA induction, the IA lesions at the right anterior cerebral (ACA)-olfactory artery (OA) bifurcation were harvested and subjected to the immunohistochemical analyses. The representative images of immunohistochemistry for CD68 (green), smooth muscle α-actin (SMA), a marker for SMCs, (red), nuclear staining by DAPI (blue) and merged images are shown. In the lower panels, the representative magnified images of immunostaining corresponding to the square in the upper panel are shown. The internal elastic lamina (IEL) is traced in white. Bars, 10 µm.

B The foam cell present in the intimal hyperplasia of IA lesions. On the 21st day after IA induction, the IA lesions at the right ACA-OA bifurcation were harvested and subjected to the serial block-face scanning electron microscopic observation. Arrow heads indicate the IEL. In the lower panels, the representative magnified images corresponding to the square are shown. Dotted line traces the cell surface and the asterisk indicates a foamy structure. Bars, 10 µm.

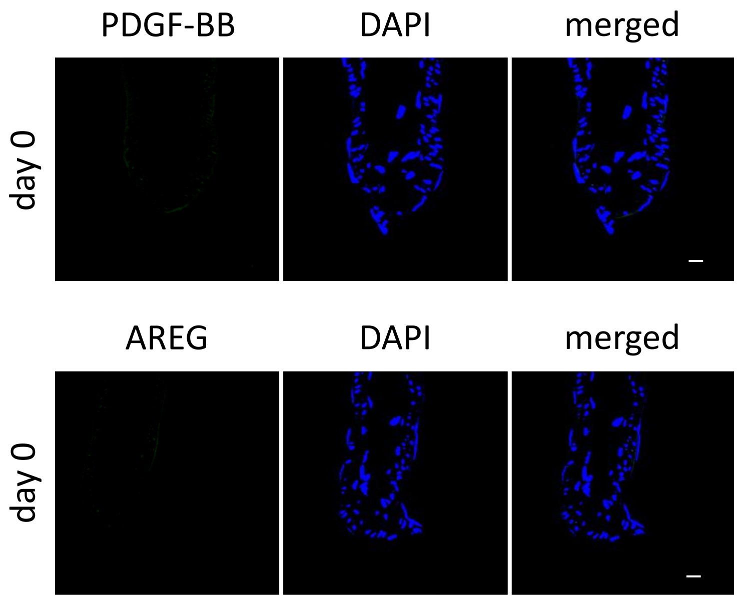

Figure S9. Absence of PDGF-BB or AREG expression in the bifurcation site without intracranial aneurysm induction.

The right anterior cerebral-olfactory artery bifurcation before intracranial aneurysm induction (day 0) was harvested and subjected to the immunohistochemical analyses. The representative images of immunohistochemistry for PDGF-BB (green in the upper panel) or Amphiregulin (AREG) (green in the lower panel), nuclear staining by DAPI (blue) and merged images are shown. Bars, 10 µm.

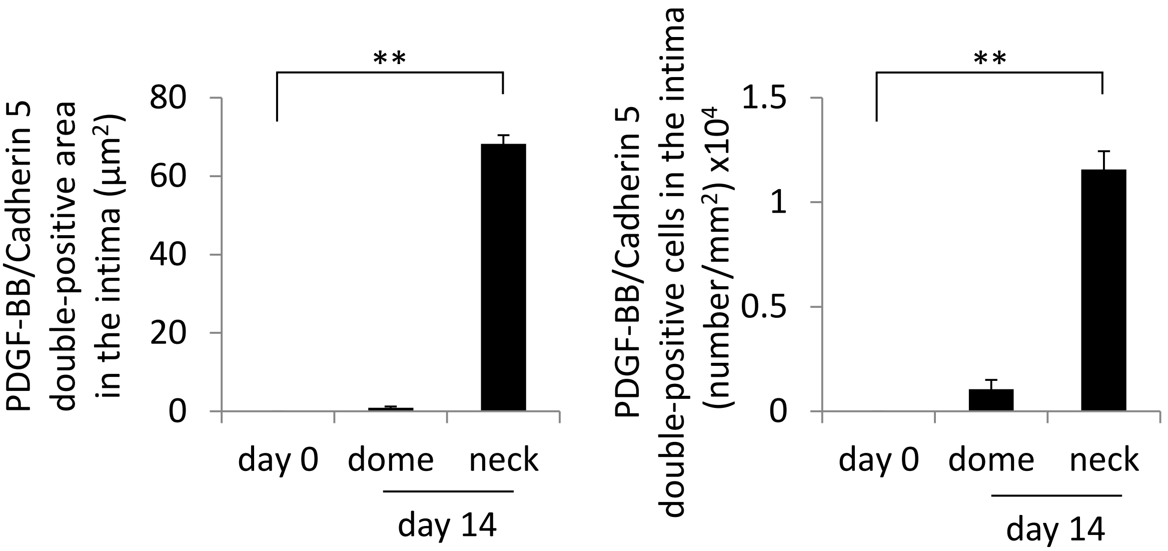

Figure S10. The quantified data of PDGF-BB-expressing Cadherin 5-positive endothelial cells in the intracranial aneurysm lesions.
Before (day 0) or on the 14th day after intracranial aneurysm (IA) induction (day 14), IA lesions at the right anterior cerebral-olfactory artery bifurcation were harvested and subjected to the immunostaining for PDGF-BB and Cadherin 5. The area of signals double positive for PDGF-BB and Cadherin 5 in immunohistochemistry in the intima and the number of PDGF-BB/Cadherin 5-double positive cells in the intima per the area of whole intracranial aneurysm lesions are shown. Data represents the mean ± SEM (n=5). Statistical analysis was done by a Kruskal–Wallis test followed by the Dunn's test. **; $p < 0.01$.

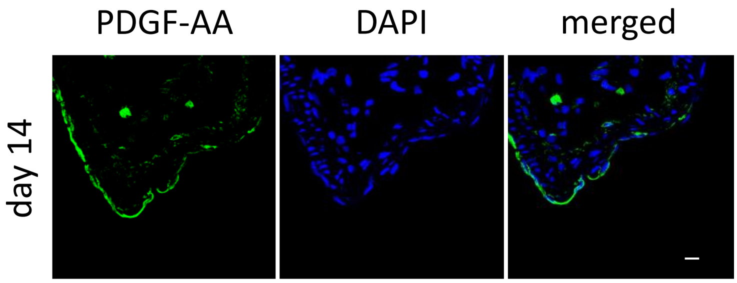

Figure S11. Expression of PDGF-AA in intracranial aneurysm lesions induced in rats.
On the 14th day after intracranial aneurysm (IA) induction (day 14), IA lesions at the right anterior cerebral-olfactory artery bifurcation were harvested and subjected to the immunostaining for PDGF-AA. The representative images of immunohistochemistry for PDGF-AA (green), nuclear staining by DAPI (blue) and merged images are shown. Bar, 10 µm.

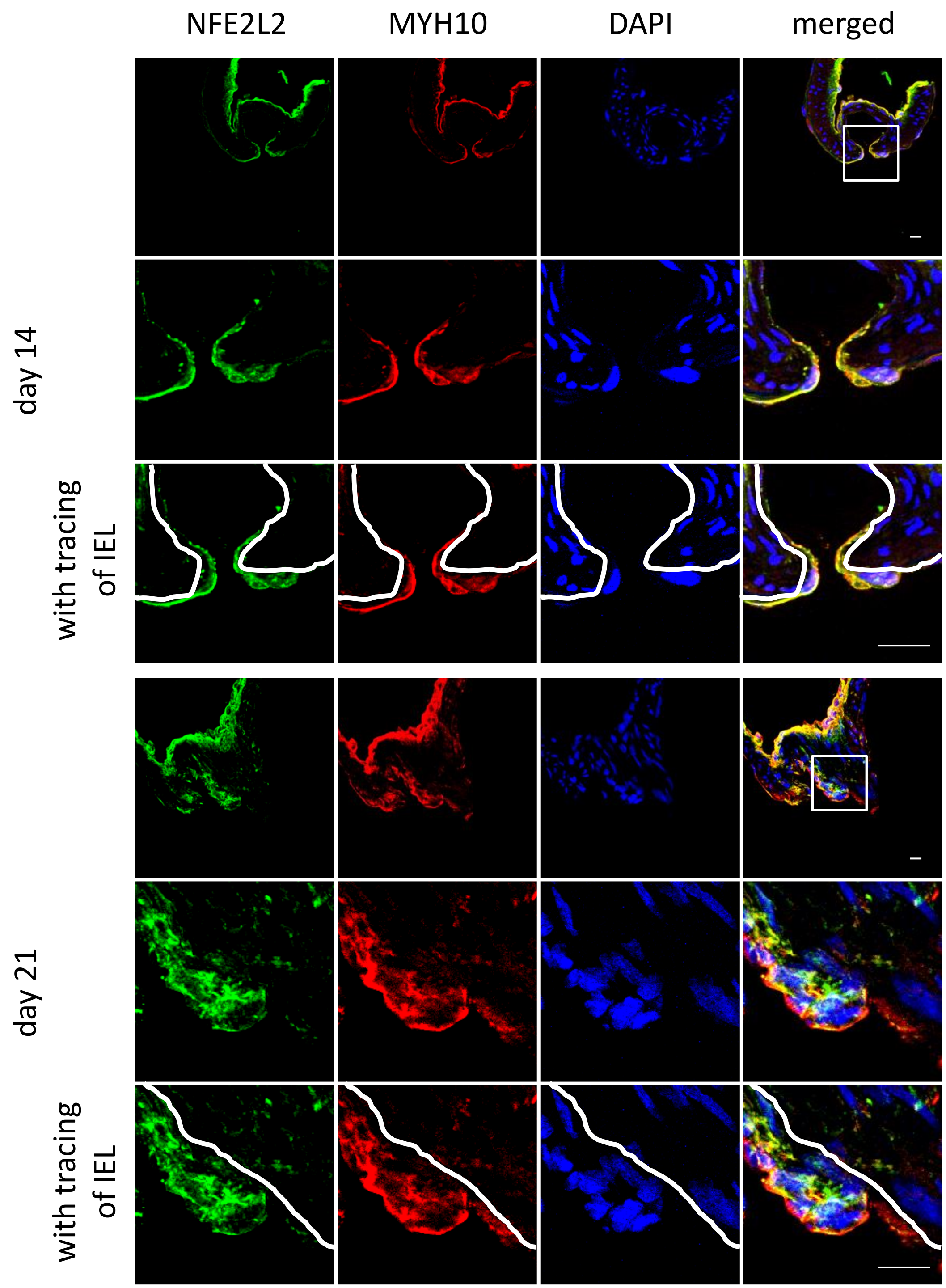

Figure S12. Expression of NFE2L2 and myosin heavy chain 10 in the intimal hyperplasia of intracranial aneurysm lesions induced in rats.
On the 14th (day 14) or 21st day (day 21) after intracranial aneurysm (IA) induction, the IA lesions at the right anterior cerebral-olfactory artery bifurcation were harvested and subjected to the immunostaining for NFE2L2 (also known as Nrf-2). The representative images of immunohistochemistry for NFE2L2 (green), myosin heavy chain 10 (MYH10), a marker for dedifferentiated smooth muscle cells, (red), nuclear staining by DAPI (blue) and merged images are shown. In the lower panels, the representative magnified images corresponding to the square in the upper panels are shown. The internal elastic lamina (IEL) is traced in white. Bars, 10 μm.

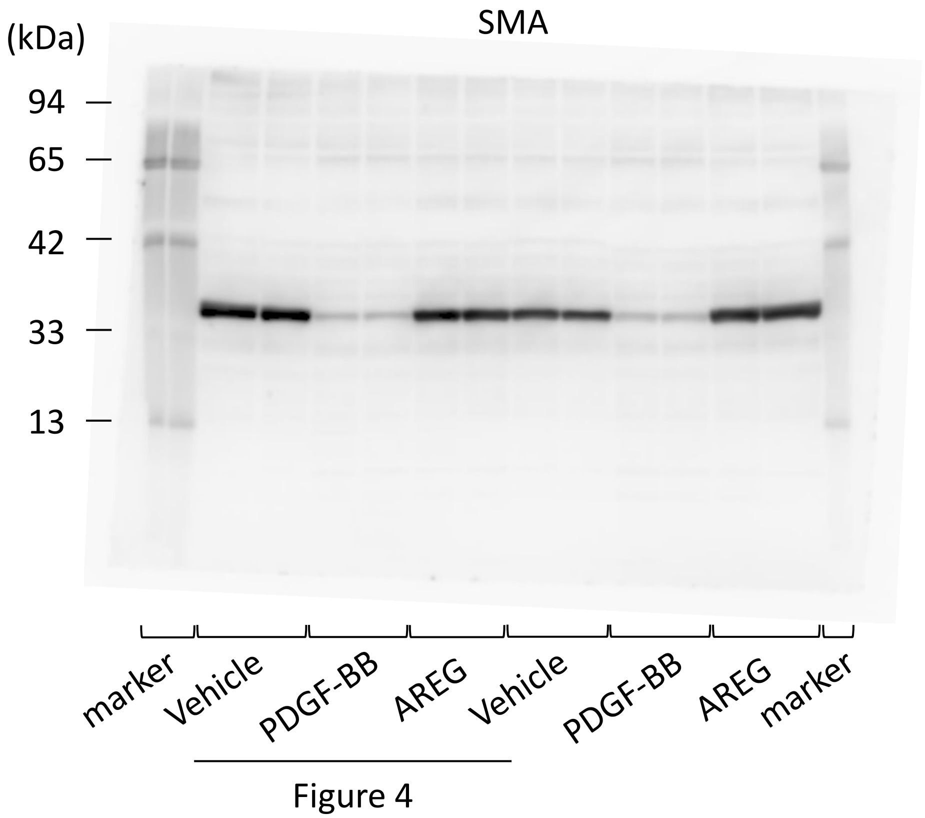

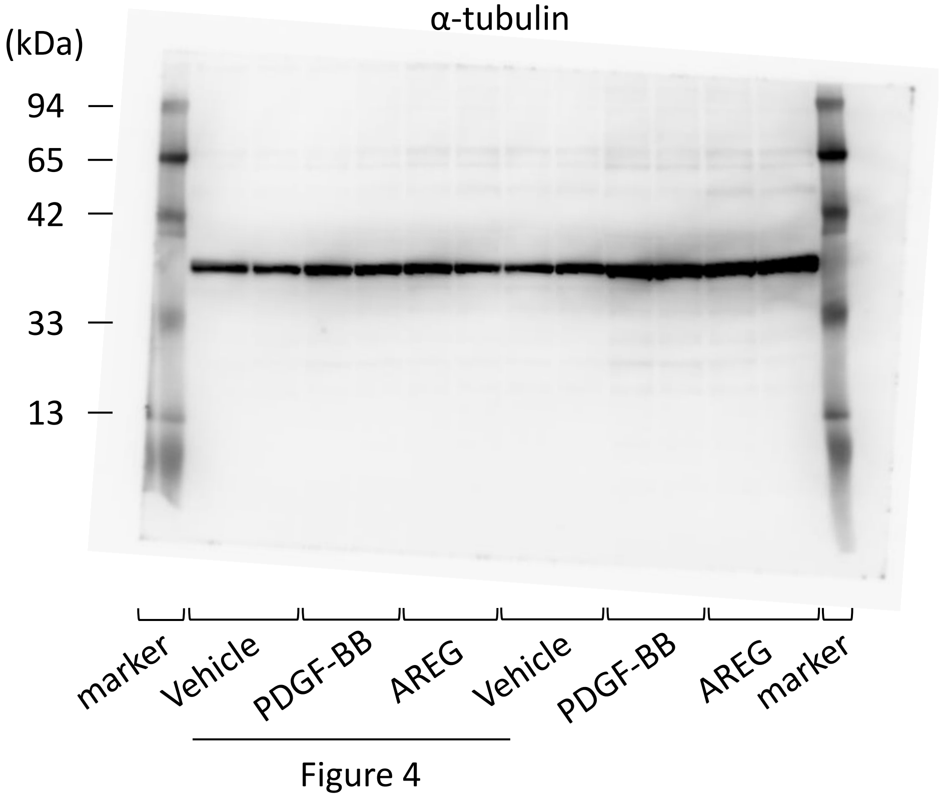

Figure S13. The raw images of western blot analyses.
The raw images of western blot analyses corresponding to the panel in Figure 4C are shown. SMA; smooth muscle α-actin.

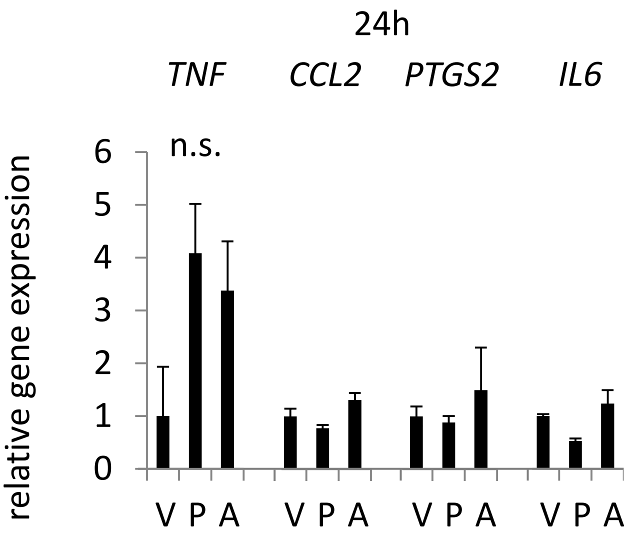

Figure S14. Effect of PDGF-BB or Amphiregulin on expression of pro-inflammatory genes in cultured smooth muscle cells.

Primary culture of smooth muscle cells were stimulated with vehicle (V), 100 ng/ml PGDF-BB (P) or 100 ng/ml Amphiregulin (A) for 24 h and expression of *TNF*, *CCL2*, *PTGS2* or *IL6* was examined by quantitative RT-PCR analysis. Data represents the mean ± SEM (n=4). Statistical analysis was done by a Kruskal–Wallis test followed by the Dunn's test. n.s.; not significant.

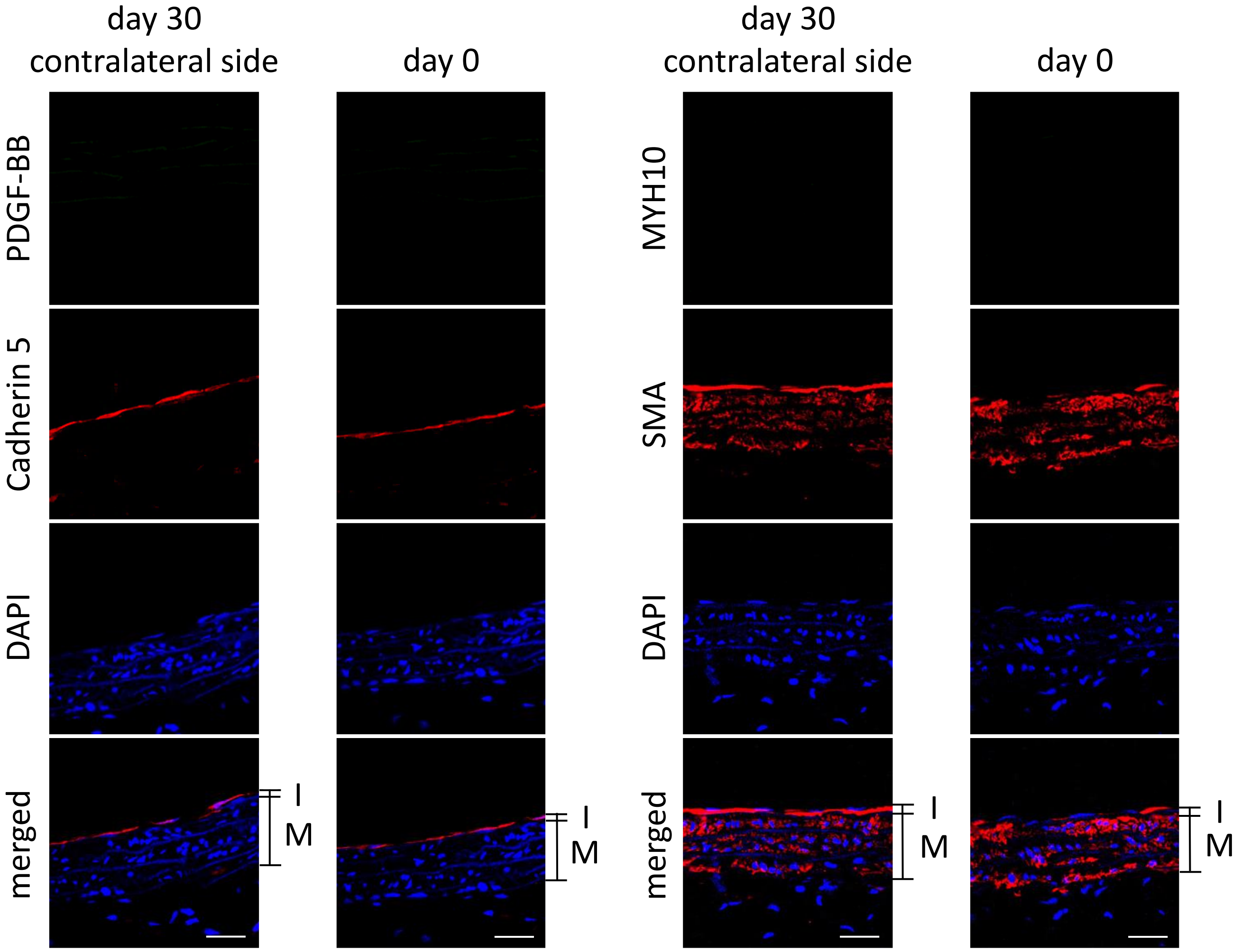

Figure S15. Absence of PDGF-BB or MYH10 expression in a carotid artery without stenosis.
The contralateral common carotid artery at 30 days after surgical manipulations to induce stenosis or before manipulations (day 0) were harvested and subjected to the immunohistochemical analyses. The representative images of immunohistochemistry for PDGF-BB (green in the left panels), myosin heavy chain 10 (MYH10, green in the right panels), Cadherin 5, a marker for endothelial cells, (red in the left panels), smooth muscle α-actin (SMA, red in the right panels), nuclear staining by DAPI (blue) and merged images are shown. I or M indicates the intima or the media, respectively. Bars, 20 μm.

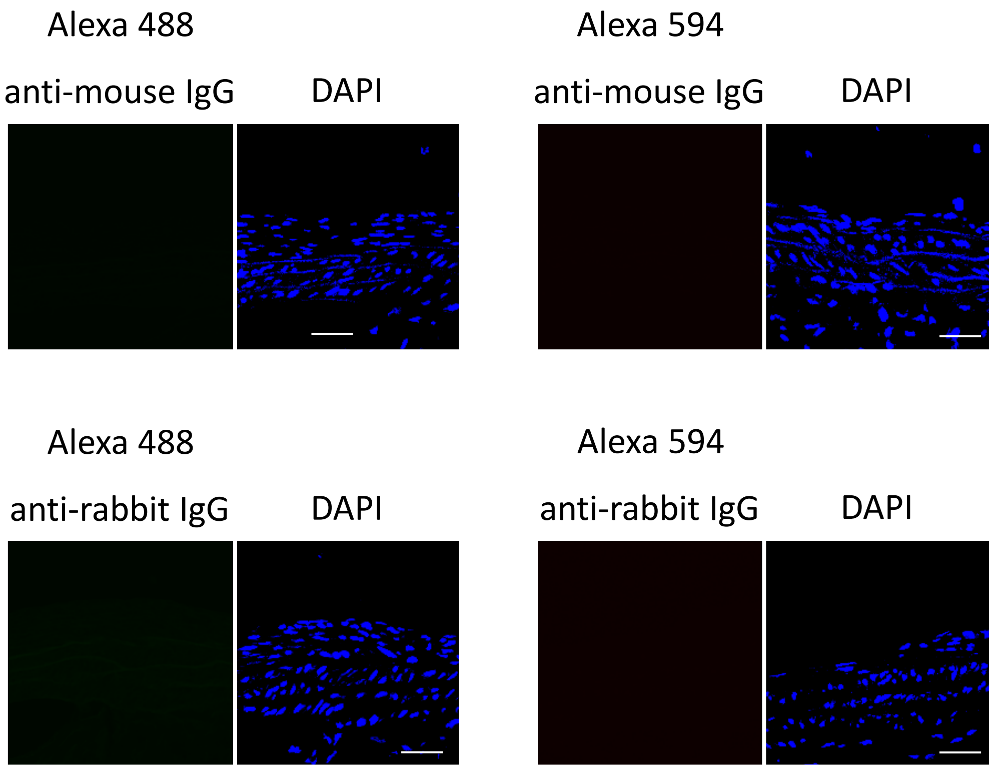

Figure S16. Immunostaining of stenotic lesions without primary antibodies as a negative control study. Stenotic lesions of a stenosis model of rats were harvested and subjected to the immunohistochemical analyses. The representative images of immunohistochemistry using only secondary antibodies, (Alexa 488-conjugated anti-mouse IgG, Alexa 488-conjugated anti-rabbit IgG, Alexa 594-conjugated anti-mouse IgG, Alexa 594-conjugated anti-rabbit IgG), are shown with nuclear staining by DAPI (blue). Bars, 10 μm.

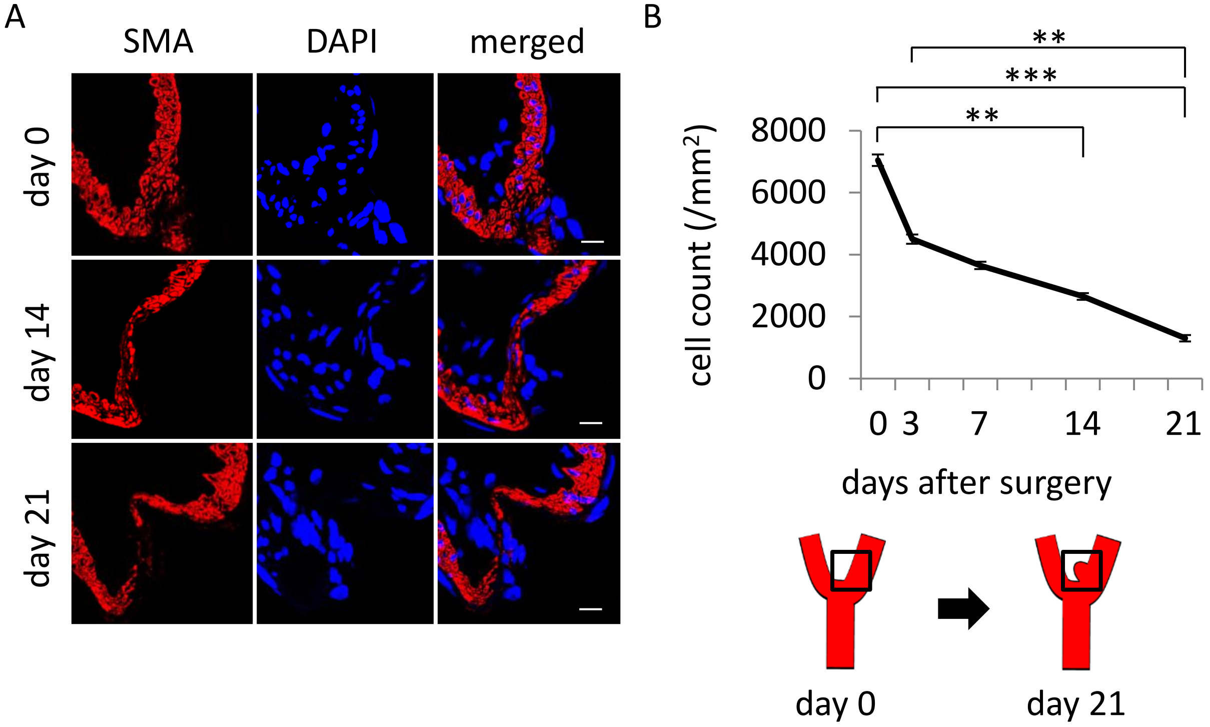

Figure S17. The loss of medial smooth muscle cells during the progression of intracranial aneurysms. **A and B** At the indicated days after intracranial aneurysm (IA) induction, the IA lesions at the right anterior cerebral-olfactory artery bifurcation were harvested and subjected to the immunohistochemical analysis. The representative images of immunohistochemistry for smooth muscle α-actin (SMA), a marker for smooth muscle cells (SMCs), (red), nuclear staining by DAPI (blue) and merged images are shown in **A**. Bars, 10 µm. The number of SMCs per unit cell area (/mm^2) was counted. Data represents the mean ± SEM in **B** (n=8). Statistical analysis was done by a Kruskal–Wallis test followed by the Dunn's test. **; p< 0.01. ***; p< 0.001. The lower panel in **B** is a schematic diagram of the arterial bifurcation where IA is induced and the square indicate the field shown in **A**.

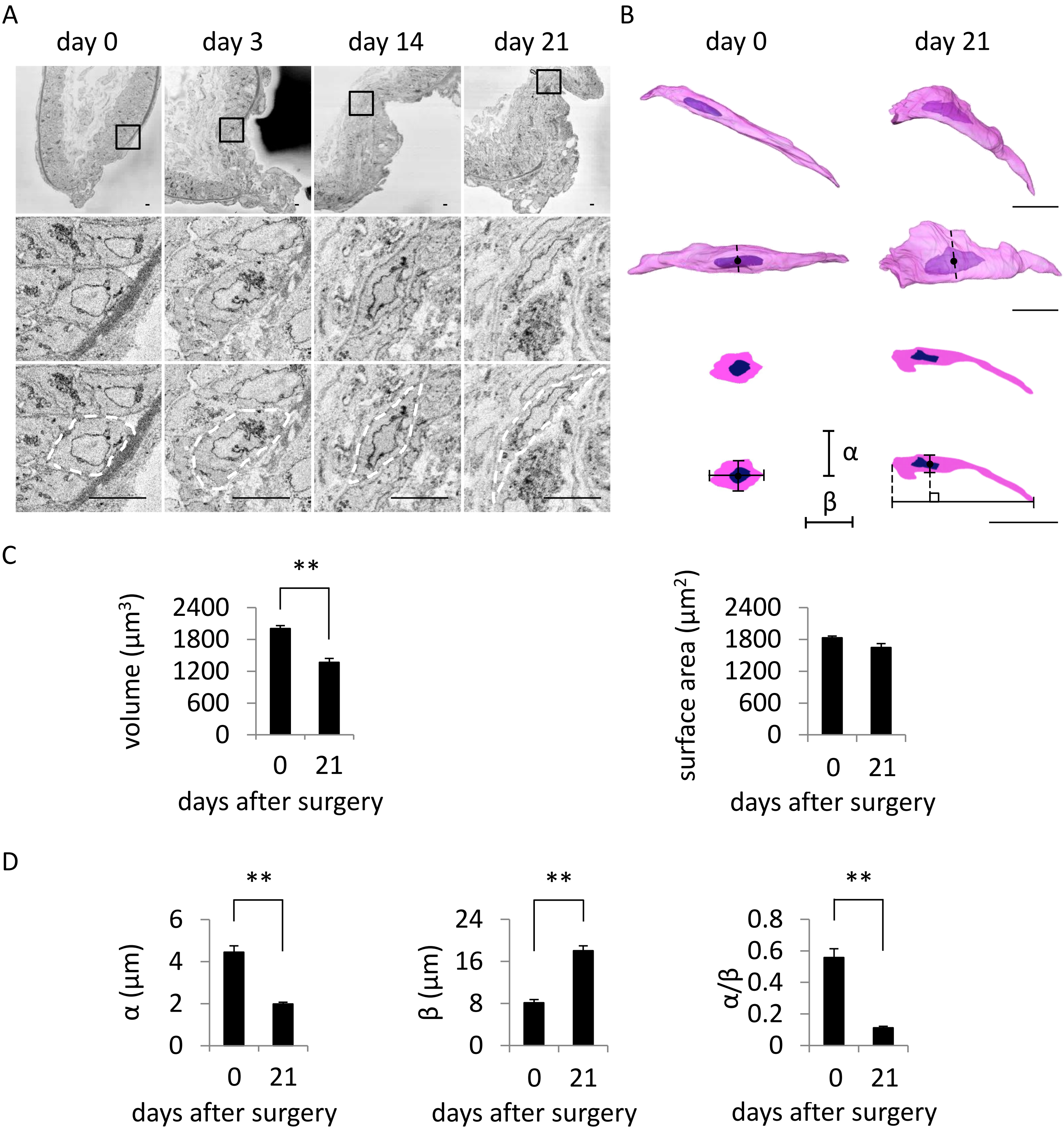

Figure S18. The morphological changes of medial smooth muscle cells during the progression of intracranial aneurysms.

A-**D** On the 3rd, 14th or 21st day after and before (day 0) intracranial aneurysm (IA) induction, the IA lesions at the right anterior cerebral-olfactory artery bifurcation were harvested and subjected to the serial block-face scanning electron microscopic (SBF-SEM) observation. The images of IA lesions acquired by SBF-SEM (**A**) and the three-dimensional reconstructed ones (**B**) are shown. Bars, 10 μm. The volume (the left panel in **C**), the surface area (the right panel in **C**), the shortest (the left panel in **D**), the longest diameter passing through the center point (the middle panel in **D**) or its ratio (the right panel in **D**) was calculated and each graph is shown. Data represents the mean ± SEM (n=6). Statistical analysis was done by a Mann–Whitney U test. **; $p < 0.01$.
